# Determining the immune environment of cutaneous T-cell lymphoma lesions through the assessment of lesional blood drops

**DOI:** 10.1038/s41598-021-98804-0

**Published:** 2021-10-04

**Authors:** Kan Torii, Yukinori Okada, Akimichi Morita

**Affiliations:** 1 Department of Geriatric and Environmental Dermatology, Nagoya City University Graduate School of Medical Sciences, Mizuho-Ku, Nagoya, 467-8601 Japan; 2 Department of Statistical Genetics, Osaka University Graduate School of Medicine, Osaka, Japan; 3 Laboratory of Statistical Immunology, Immunology Frontier Research Center (WPI-IFReC), Osaka University, Suita, Japan; 4 Integrated Frontier Research for Medical Science Division, Institute for Open and Transdisciplinary Research Initiatives, Osaka University, Osaka, Japan

**Keywords:** Skin cancer, Lymphoma

## Abstract

Detailed analysis of the cells that infiltrate lesional skin cannot be performed in skin biopsy specimens using immunohistochemistry or cell separation techniques because enzyme treatments applied during the isolation step can destroy small amounts of protein and minor cell populations in the biopsy specimen. Here, we describe a method for isolating T cells from drops of whole blood obtained from lesions during skin biopsy in patients with cutaneous T-cell lymphoma. Lesional blood is assumed to contain lesional resident cells, cells from capillary vessels, and blood overflowing from capillary vessels into the lesion area. The lesional blood showed substantial increases in distinct cell populations, chemokines, and the expression of various genes. The proportion of CD8^+^CD45RO^+^ T cells in the lesional blood negatively correlated with the modified severity-weighted assessment tool scores. CD4^+^CD45RO^+^ T cells in the lesional blood expressed genes associated with the development of cancer and progression of cutaneous T-cell lymphoma. In addition, CD8^+^CD45RO^+^ T cells in lesional blood had unique T-cell receptor repertoires in lesions of each stage. Assessment of lesional blood drops might provide new insight into the pathogenesis of mycosis fungoides and facilitate evaluation of the treatment efficacy for mycosis fungoides as well as other skin inflammatory diseases.

## Introduction

The tissue environment surrounding skin lesions has an important role in skin disease. Harvesting cells from skin lesions can be time-consuming and challenging, however, due to the considerable cell and protein loss caused by tissue degradation. Various techniques are thus used to analyze skin lesion cells and the surrounding environment, including multiphoton excitation microscopy^1^, dermal open-flow microperfusion^2^, and immersion of skin samples in medium to extract cells^3^. The cell isolation processes required for these techniques, however, may lead to the loss of critical information. For example, while lymphocytes can be isolated from skin tissue obtained by punch biopsy, the small amount of tissue contains too few cells and thus provides limited information.

Alternatively, lesional blood samples could provide valuable information about the surrounding environment, including the levels and types of cytokines and inflammatory cells, without the need for enzyme treatment. In fact, a previous study reported the successful use of sera from peripheral blood and blood obtained from psoriasis lesions to assess the skin lesion environment^4^. Lesional blood samples might therefore be useful for isolating and analyzing lesional cellular components and serum.

Skin biopsies are regularly obtained to diagnose and assess treatment efficacy in patients with cutaneous T-cell lymphoma (CTCL). Diagnosis of CTCL is relatively difficult^5^, however, and effective treatments are not yet clearly established. Better methods that allow for rapid isolation and analysis of resident and systemic pathogenic T cells and effector T cells are necessary to facilitate diagnosis and develop effective treatments. Mycosis fungoides (MF), the most common CTCL, is considered to be a low-grade T-cell lymphoma^6^. The premycotic and mycotic phases can last several years, but in some cases the disease progresses very rapidly^7^. Due to the relatively low awareness and diagnostic difficulties of MF, many patients seek dermatologic consultation for the first time after their condition has already progressed to the mycotic or tumor stage. This delay in diagnosis may lead to tumor formation, ulceration, leukemic transformation, visceral invasion, and death within a few months. The histologic findings depend on the disease stage. In the erythema stage (stage I), characteristic features include epidermal hyperplasia, lymphoid exocytosis, and band-like lymphoid infiltration in the superficial dermis. In the plaque stage (stage II), Pautrier’s microabscesses are often observed. In the tumor stage (stage III), tumor cells infiltrate the nodular lesions and proliferate with necrosis, leading to ulcer formation in tumorous lesions^7^. Cells infiltrating MF lesions have an α/β memory T-helper phenotype. In the advanced tumor stage, T-cell markers may be lost and a T-cytotoxic phenotype is observed. Several biopsies of MF lesions obtained from a single patient within a short period of time may reveal various phenotypes^8^. The effector cells that infiltrate MF lesions, however, remain unclear.

Lesional blood could be useful for obtaining more detailed information about the phenotype and immune microenvironment in MF. In the present study, we analyzed drops of lesional whole blood, i.e., a very small amount of blood obtained from the lesion site during the skin lesion biopsy, to assess the types of T cells induced in patients with MF while concurrently analyzing RNA expression. We were also able to determine the functions of the infiltrating T cells with high accuracy. The development of an effective method of analyzing lesional blood drops may also be useful for evaluating other skin inflammatory diseases, such as atopic dermatitis and psoriasis, as well as the efficacy of their treatments.

## Results

### CD4^+^/CD8^+^ T cells are successfully isolated from a small amount of lesional blood

**Figure 1:**
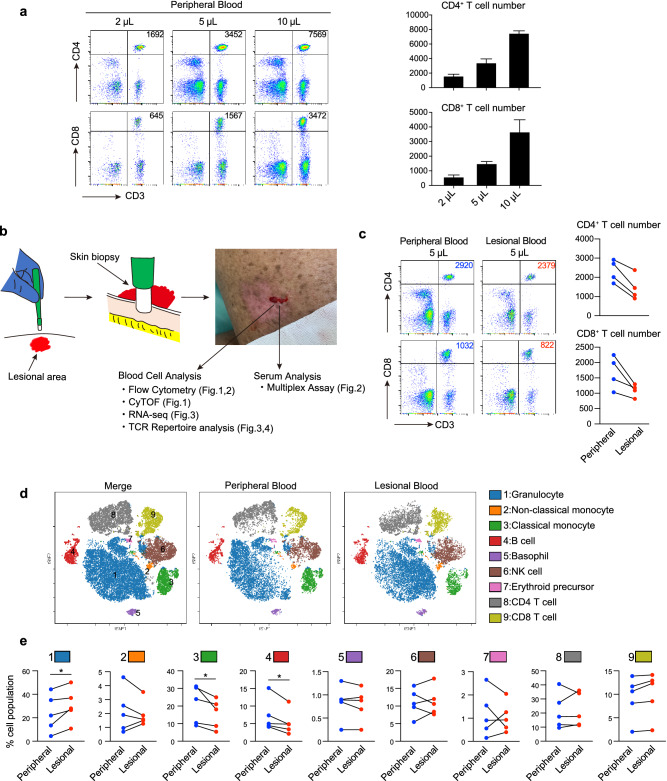
Cell populations differ between peripheral and lesional blood in patients with mycosis fungoides (MF). (**a**) Flow cytometry analysis of CD4^+^ and CD8^+^ T cells from 2, 5, and 10 μL peripheral blood for the detection of T cells in small amounts of blood. The number in the upper right corner represents the number of CD4^+^ and CD8^+^ T cells. Mean (+ standard deviation) numbers of CD4^+^ and CD8^+^ cells (n = 3) are shown in the bar graph (right). (**b**) Lesional blood was collected during a skin biopsy. Peripheral and lesional blood was obtained from the patient’s arm and erythematous lesion, respectively, on the same day. The collected blood cells and serum were used for the subsequent experiments. (**c**) Flow cytometry of CD4^+^ and CD8^+^ T cells obtained from 5 μL of peripheral and lesional blood. Numbers of CD4^+^ and CD8^+^ T cells in 5 μL of peripheral and lesional blood are shown (right, n = 4). (**d**) Representative mass cytometry analysis of peripheral and lesional blood from a patient with MF through viSNE analysis using Cytobank. The color of each dot represents the immune cell subset. (**e**) The proportion of each cell population in peripheral and lesional blood obtained from patients with MF (n = 5) quantified by mass cytometry. A paired *t*-test was used for the statistical analysis. * *P* < 0.05.

We obtained 200 to 300 µL of lesional blood from the wound site resulting from the skin biopsy. The cells were separated from the sample using a cell sorter. Approximately 3000 CD4^+^ T cells and 1000 CD8^+^ T cells were successfully isolated from 5 μL of peripheral blood (Fig. 1a). Although the lesional blood contained slightly fewer cells than the peripheral blood, we were able to collect a sufficient number of cells from lesional blood for the analyses (Fig. 1b,c). Cytometry by time-of-flight (CyTOF) revealed that the lesional blood contained more granulocytes, and fewer monocytes and B cells than the peripheral blood (Fig. 1d,e). These findings indicate that the cell populations in lesional blood might differ from that in peripheral blood. Isolated cells and sera from lesional blood were therefore further analyzed.

### CD8^+^CD45RO^+^ T-cells in lesional blood negatively correlate with the mSWAT score

**Figure 2:**
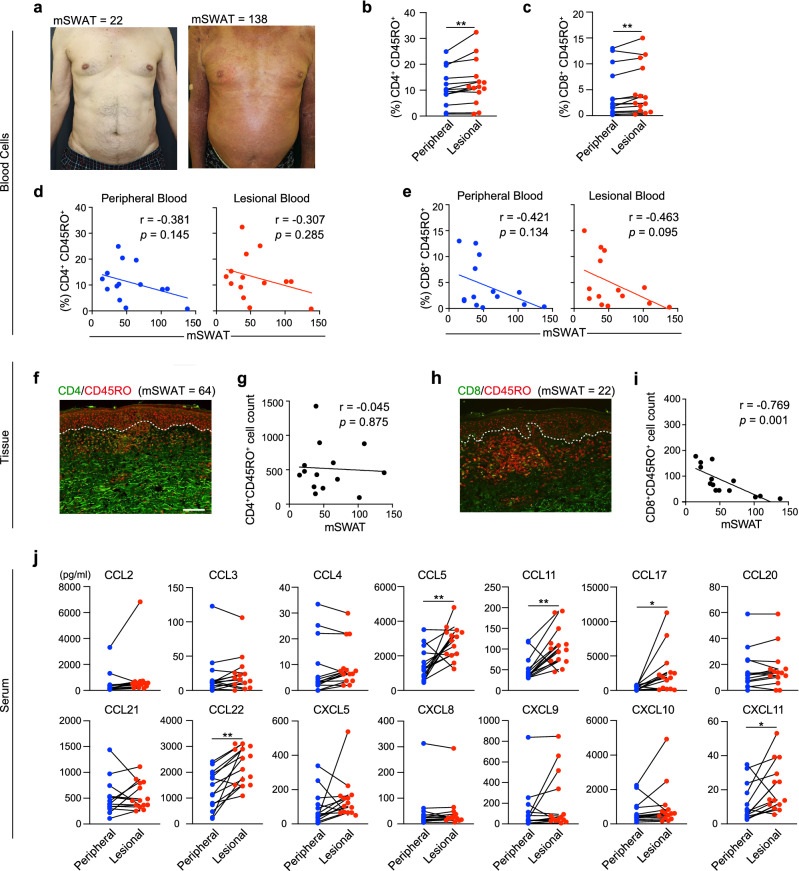
CD8^+^CD45RO^+^ cells negatively correlate with the mSWAT score in patients with MF. (**a**) Typical clinical features of MF lesional skin with an mSWAT score of 22 (left) and an mSWAT score of 138 (right). (**b,c**) The percentage of CD4^+^CD45RO^+^ and CD8^+^CD45RO^+^ T cells in lesional blood was higher than that in peripheral blood. Paired *t*-test. ** *P* < 0.01. (**d,e**) Correlation of CD4^+^CD45RO^+^ T cells and CD8^+^CD45RO^+^ T cells in peripheral blood and lesional blood with the mSWAT score evaluated by Pearson correlation coefficient analysis (n = 14). (**f–i**) Representative immunofluorescence staining of CD4/8 (green) and CD45RO (red) in MF lesional skin. Scale bar = 100 μm. Correlation of CD4^+^CD45RO^+^ cells and CD8^+^CD45RO^+^ cells with mSWAT (n = 14) evaluated by Pearson correlation coefficient analysis. Quantification of the number of cells per visual field was performed using the Hybrid Cell Count BZ-H4C analyzer software. Data were statistically analyzed using the Pearson correlation test (2-tailed). **j**) Results of multiplex chemokine bead assay using sera from peripheral and lesional blood (n = 14). Paired *t*-test. * *P* < 0.05, ** *P* < 0.01.

We obtained 14 biopsy specimens from the lesional skin of MF patients for immunohistochemical analysis (Table 1). All MF patients were assessed using the modified severity-weighted assessment tool (mSWAT), and skin biopsies were obtained upon admission (Fig. 2a). Although in the mass cytometry analysis of the initial 4 samples, the proportions of CD4^+^ and CD8^+^ T cells did not differ significantly between lesional blood and peripheral blood (Fig. 1e), flow cytometry analysis of the 14 additional samples using a paired t-test revealed that lesional blood contained a significantly greater proportion of CD4^+^CD45RO^+^ and CD8^+^CD45RO^+^ T cells (Fig. 2b,c). Furthermore, the proportion of CD8^+^CD45RO^+^ T cells in the lesional and peripheral blood negatively correlated with the mSWAT score (Fig. 2e). The proportion of CD4^+^CD45RO^+^ T cells weakly inversely correlated with the mSWAT score (Fig. 2d). Thus, assessment of lesional blood drops might reveal the phenotypic details of infiltrating cells.

**Table 1:** Patient background.

Pt	Age (yr)	Sex	Disease	Stage	mSWAT	CD8/45RO (%, PB)	CD8/45RO (%, LB)	Tissue CD8/45RO	CyTOF	RNA-seq	TCR repertoire	Treatment
1	83	M	MF	IB	44	10.4	11.2	45				Chemo + PUVA
2	68	M	MF	IB	49	0.18	0.5	45				Chemo
3	61	F	MF	IIIA	64	3.25	3.54	44				Chemo + PUVA
4	88	F	MF	IB	38	7.69	9.18	8*9*		CD8/45RO	CD8/45RO	PUVA
5	71	F	MF	IB	102	3.12	4.03	19	○			Chemo + PUVA
6	82	M	MF	IB	40	0.65	0.72	67	○			Chemo
7	56	F	MF	IB	36	2.22	2.33	71	○			Chemo + PUVA
8	70	M	MF	IIIA	138	0.34	0.26	13				Chemo + PUVA
9	78	F	MF	IB	22	1.71	3.81	135	○			Chemo + PUVA
10	71	M	MF	IIB	109	0.76	0.97	23	○		CD8/45RO	Chemo + PUVA
11	64	F	MF	IB	70	2.3	2.4	82			CD4/45RO	Chemo + PUVA
12	72	F	MF	IB	39	12.6	11.8	167		CD4/45RO		Chemo + PUVA
13	78	M	MF	IA	15	13	15	178				PUVA
14	72	M	MF	IB	22	1.5	1.9	154			CD8/45RO	Chemo + PUVA
15	45	F	MF	IA	14	1.88	2.3				CD8/45RO	Chemo + PUVA
16	67	M	MF	IIIA	94	1.16	1.81			CD4/45RO		Chemo
17	45	M	MF	IB	26	1.71	1.7			CD4/45RO		Chemo + PUVA
18	70	M	MF	IB	35	3.42	3.44			CD8/45RO		Chemo + PUVA
19	34	F	MF	IB	40	1.26	1.45			CD8/45RO		Chemo + PUVA

All MF patients are CD4^+^ MF and underwent skin biopsies on admission treatment. *MF* mycosis fungoides, *Chemo* chemotherapy (bexarotene), *PUVA* psoralen and ultraviolet A.

### CD8^+^CD45RO^+^ T-cells infiltrate MF lesions and negatively correlate with CTCL pathogenesis

The CD8^+^ tumor-infiltrating lymphocyte levels in patients with MF positively correlate with an improved survival rate and exert an antitumor effect^9^. Therefore, increased levels of CD8^+^ T cells in CTCL serve as a promising criterion for predicting patient survival and supporting treatment decisions, and inclusion of patients in randomized controlled trials^10^. Moreover, the partial activation of CD8^+^ cytotoxic T cells present in CTCL and their positive correlation with a better prognosis suggest that they have an important role in the antitumor response^11^. Tissue specimens showed a negative correlation between the mSWAT score and CD8^+^CD45RO^+^ T cells with less infiltration of effector CD8^+^ T cells in advanced cases (Fig. 2h,i). In contrast, CD4^+^CD45RO^+^ T cells in tissue specimens did not correlate with mSWAT scores (Fig. 2f,g), consistent with the results from the lesional blood. These infiltrating cells in tissue specimens, however, cannot be easily assessed by immunohistochemistry alone.

### Chemokine profiles differ between lesional blood and peripheral blood

In sera simultaneously isolated from peripheral and lesional blood samples, the levels of chemokines such as CCL5, CCL11, CCL17, CCL22, and CXCL11 were significantly increased in the lesional blood compared with the peripheral blood (Fig. 2j). The increases in these chemokines are specific to MF lesions. CCL5, CCL17, and CCL22 are derived from keratinocytes; CCL22 and CXCL11 are derived from endothelial cells; and CCL11 is derived from macrophages in MF lesions^12^. In particular, levels of the CCR4 ligands CCL17 and CCL22 are upregulated in the epidermis and serum of patients with MF^13–15^. Malignant T cells expressing CCR4 are recruited by CCL17 and CCL22^14^. We found that sera from lesional blood revealed a specific chemokine environment for MF. The results demonstrated that the chemokine profile of lesional blood differs from that of peripheral blood, indicating that lesional blood drops are sufficient for obtaining a detailed chemokine profile of the lesion environment.

### CD4^+^CD45RO^+^ T cells and CD8^+^CD45RO^+^ T cells from lesional and peripheral blood differ in RNA sequence and T-cell receptor repertoire analyses

**Figure 3:**
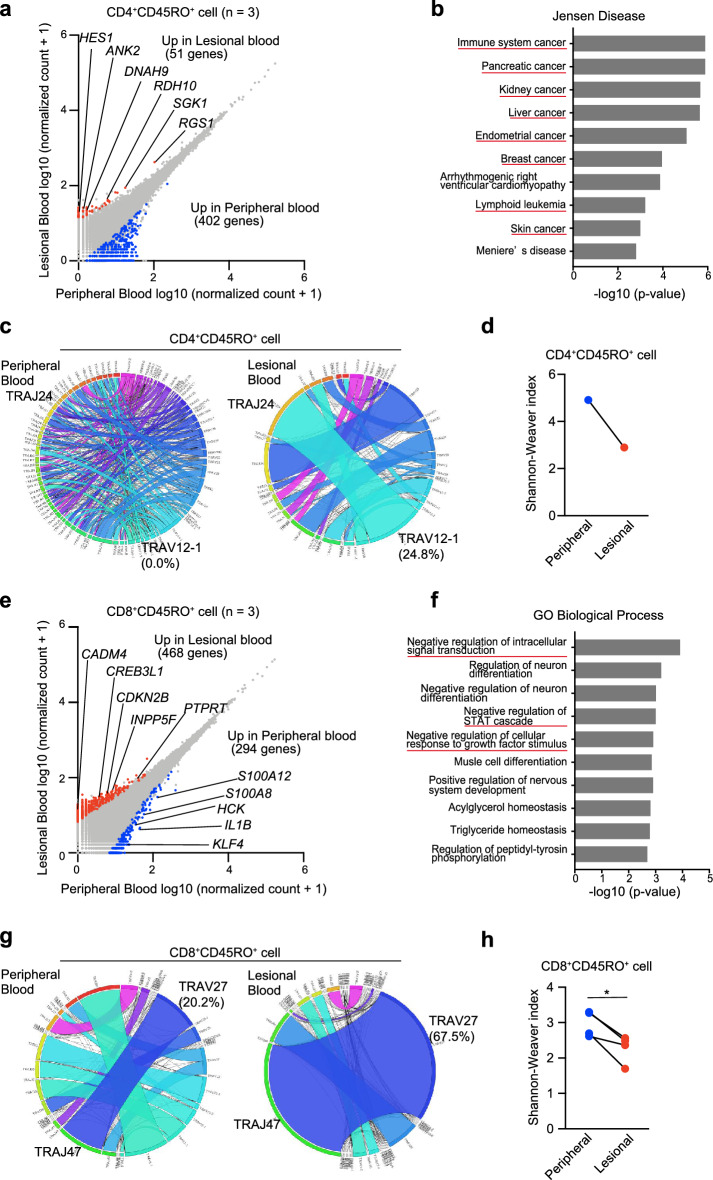
TCR repertoire of lesional blood is skewed, and expression of genes differs between lesional and peripheral blood. (**a**) Gene expression analysis through RNA-seq in CD4^+^CD45RO^+^ T cells of peripheral and lesional blood from 3 patients (Cases 12, 16, and 17). Scatter plots show the expression values of every annotated gene. Blue and red dots indicate significant upregulation of CD4^+^CD45RO^+^ T cells in peripheral and lesional blood, respectively. (**b**) Ten top-ranked terms from the Jensen Diseases library of Enrichr for genes upregulated in CD4^+^CD45RO^+^ T cells of lesional blood. (**c**) Circos plots of frequencies of Vα and Jα gene usage and combinations of productive sequences in CD4^+^CD45RO^+^ T cells of peripheral and lesional blood. The band widths represents the frequency of each VJ pair. (**d**) Diversity of the TCR repertoire in CD4^+^CD45RO^+^ T cells of peripheral and lesional blood was evaluated using the Shannon–Weaver Index. (**e**) RNA-seq in CD8^+^CD45RO^+^ T cells of peripheral and lesional blood from 3 patients (Cases 4, 18, and 19). (**f**) Ten top-ranked gene ontology terms of biologic processes for genes upregulated in CD8^+^CD45RO^+^ T cells of lesional blood. (**g**) Circos plots of CD8^+^CD45RO^+^ T cells of peripheral and lesional blood. (**h**) Shannon–Weaver Index of CD8^+^CD45RO^+^ T cells of peripheral and lesional blood.

We isolated CD4^+^CD45RO^+^ and CD8^+^CD45RO^+^ T cells from lesional and peripheral blood (n = 3) using a FACS Melody sorter (Becton Dickinson). RNA sequencing (RNA-seq) of the isolated cells was performed to analyze the transcriptome. CD4^+^CD45RO^+^ T cells in the lesional blood highly expressed genes relating to cancer progression and CTCL pathogenesis: *RGS1*, *RDH10*, *HES1*, *DNAH9*, *ANK2*, and *SGK1* (Fig. 3a)^16–25^. To further confirm the increases in the 51 highly expressed genes, the genes were enriched in the Jensen Disease library of Enrichr. Enrichr showed that the diseases were related to cancer, including “skin cancer” and “lymphoid leukemia” (Fig. 3b). All differentially expressed genes are listed in Table S1. Although we performed T-cell receptor (TCR) repertoire analysis only for 1 case, CD4^+^CD45RO^+^ T cells in the lesional blood exhibited a unique TCR repertoire, showing reduced diversity compared with the TCR repertoire in peripheral blood (Fig. 3c,d). Specific polymorphisms in *TP53* and *STAT3* are associated with CTCL malignancy^26^. In Cases 16 and 17, the frequency of *TP53* polymorphism c.C98G (rs1042522) in CD4^+^CD45RO^+^ T cells was higher in the lesional blood than in the peripheral blood (Table S2). No *TP53* polymorphism was detected in Case 12, and *STAT3* mutations c.A1936T and c.A1480T were detected only in the lesional blood.

On the other hand, CD8^+^CD45RO^+^ T cells highly expressed the following representative genes in lesional blood samples: *CDKN2B*, *PTPRT*, *CREB3L1*, *CADM4*, and *INPP5F* (Fig. 3e). These genes encode tumor suppressor proteins^27–31^ and activate the expression of genes encoding cell cycle inhibitors, including p21^32^. Gene ontology analysis using 468 genes was performed in CD8^+^CD45RO^+^ T cells from MF samples. Among the 10 most enriched biologic processes determined using Metascape in CD8^+^CD45RO^+^ T cells isolated from MF samples, the top 3 were “negative regulation of intracellular signal transduction”, “negative regulation of STAT cascade”, and “negative regulation for cellular response to growth factor stimulation” (Fig. 3f). A previous study reported that effector cells (CD8^+^CD45RO^+^ T cells) express exhausted phenotypes^33^. Our findings confirmed that CD8^+^CD45RO^+^ T cells from lesional blood negatively regulate cellular responses. On the other hand, genes related to inflammation (*S100A12*, *S100A8*, *HCK*, *IL1B*, and *KLF4*) are increased in peripheral blood^34–37^. The TCR repertoire of CD8^+^CD45RO^+^ T cells was skewed in lesional blood (erythema area; n = 4) compared with that in peripheral blood (Fig. 3g,h). These expanded CD8^+^CD45RO^+^ T cells might be tumor antigen-specific but incapable of suppressing CTCL cells.

### Stage progression and skewed TCR repertoires in CD8^+^CD45RO^+^ T cells

**Figure 4:**
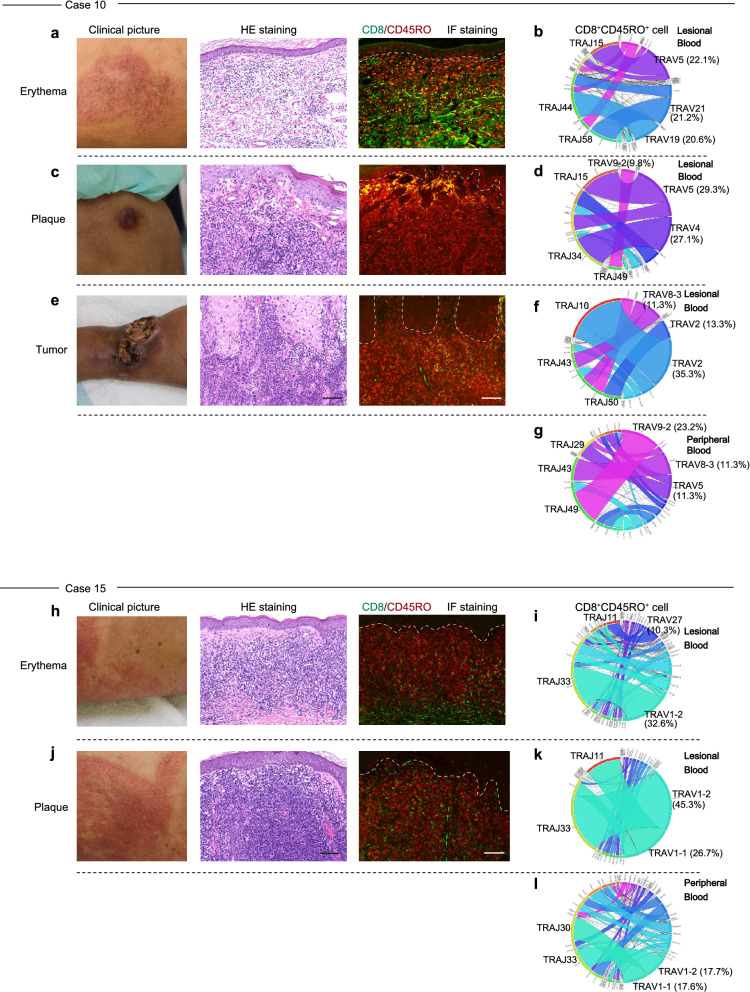
CD8^+^CD45RO^+^ TCR repertoire differs depending on the stage of MF. (**a,c,e**) Hematoxylin and eosin (HE) staining and immunofluorescence (IF) staining of 3 types of skin lesions (erythema, plaque, and tumor) from Case 10. CD8 (green) and CD45RO (red) are shown in the immunofluorescence staining image. Collagen is stained green, and red blood cells are stained red. Scale bar = 100 μm. (**b,d,f,g**) Circos plots of frequencies of Vα and Jα gene usage and combinations for CD8^+^CD45RO^+^ T cells in lesional blood from erythema, plaque, and tumor stages. The Circos plot at the bottom (**g**) is of cells obtained from peripheral blood. (**h–l**) Erythema and plaque of the same skin lesion from case 15. Same experiment as shown in panels (**a–g**).

Biopsy specimens and lesional blood were collected from lesions at each stage (erythema, plaque, and tumor) from the same patient. A certain number of CD8^+^CD45RO^+^ cells was found in the erythema areas, but few were found in the plaque and tumor tissues in Case 10 (Fig. 4a,c,e; immunofluorescence staining). We isolated CD8^+^CD45RO^+^ T cells from the lesional blood and peripheral blood separately. The isolated cells were subjected to TCR repertoire analysis. The CD8^+^CD45RO^+^ T cells in the lesional blood showed unique TCR repertoires in lesions of each stage (Fig. 4b,d,f,g). We assume that CD8^+^CD45RO^+^ cells in lesional blood would recognize a tumor antigen in tumor and plaque tissue. A previous report indicated that malignant T-cell clones exhibit heterogeneity between skin lesions in the same patient^38^. Our results are consistent with previous findings that neoplastic T-cell clones vary in skin lesions. Furthermore, different TCR repertoires were present in tumor-stage lesions of the same patient (Fig. 4f), which may result from the generation of different neoplastic T-cell clones for the growth of tumor lesions. In another patient (Case 15), a biopsy was performed from an adjacent lesion that developed erythema and plaque. CD8^+^CD45RO^+^ cells were found in both the erythema and plaque areas (Fig. 4h,j), and repertoire analysis showed that the same TCRs (TRAV1-2–TRAJ33) were increased (Fig. 4i,k,l). These findings indicate that CD8^+^ T cells with different TCR repertoires are directed toward each skin lesion, which is expected given the heterogeneity of malignant T cells in skin lesions.

## Discussion

The present study revealed that the composition of lesional blood differs from that of peripheral blood, and lesional blood appears to reflect the condition of the skin's immune environment in lesional areas of MF patients. Many techniques are used to analyze the cells and tissue environments of skin lesions, and assessment of lesional blood drops is a feasible method. Although assessment of lesional blood drops would not obviate the need for skin biopsy due to the different results, analysis of lesional blood could provide valuable information about infiltrating cells and the separated serum. This technique can also be used to maximize the retrieval of intact cells and blood when skin biopsy is performed or a tumor is resected. We assume that lesional blood would contain lesional resident cells, cells from capillary vessels, and blood overflowing from capillary vessels, allowing us to identify a unique pattern of cell populations and chemokines in the lesional blood.

Mass cytometry analysis showed differences in the cell populations between lesional and peripheral blood, and the chemokine assay revealed increases in specific chemokines in lesional blood. The different types of cells in the lesional blood may be due to the induction of chemokines. Memory T cells, CD45RO^+^ T cells, were frequently observed in lesional blood, suggesting that these cells are involved in the lesion. Chemokines that are associated with MF were particularly abundant in lesional blood. CCL17 and CCL22 are ligands for CCR4, and these chemokines are strongly associated with the pathogenesis of MF^13,14^. Although we have not yet collected lesional blood from healthy areas, we expect to clarify the detailed pathogenesis of MF by comparing lesional blood from healthy and lesional areas in the same patient.

RNA-seq of CD4^+^CD45RO^+^ T cells and CD8^+^CD45RO^+^ T cells suggested that lesional blood contains malignant T cells and effector T cells, respectively. This notion was supported by the skewed TCR repertoire in lesional blood (Fig. 3c,g). In the present study, CD4^+^CD45RO^+^ T cells from the lesional blood highly expressed genes relating to cancer progression and CTCL progression. Among the differentially expressed genes in this study, expression of *SGK1*, *HES1*, and *RDH10,* which were identified in other studies conducting single cell RNA-seq (scRNA-seq) of CTCL skin tumors^24,25^, was increased. Although bulk RNA-seq was performed in this study, these genes were also identified in CD4^+^CD45RO^+^ T cells in lesional blood (Fig. 3a). It may thus be possible to isolate lymphocytes recruited by chemokines or present in the lesion area from lesional blood. A larger number of differentially expressed genes was detected in the CD4^+^CD45RO^+^ cells of peripheral blood because cells in the lesional blood vary between lesions and stages. In addition, there are different types of malignant cells^39^, which may be why fewer differentially expressed genes were detected in the CD4^+^CD45RO^+^ cells of lesional blood (Fig. 3a). CD8^+^CD45RO^+^ T cells in the lesional blood had a less inflammatory transcriptome than CD8^+^CD45RO^+^ T cells in the peripheral blood. Effector T cells express exhausted phenotypes characterized by the expression of the *PD-1*, *ICOS*, *TIM-3*, *LAG-3*, and *CTLA-4* markers in lesional skin^33^. Although expression of these genes was not increased, weak expression of genes related to the inflammatory system and cell division suggest that CD8^+^CD45RO^+^ T cells in the lesional blood are a type of exhausted T cell^40^.

The clones of malignant cells are different in each skin lesion in patients with MF^41^. In the present study, we collected CD8^+^CD45RO^+^ cells from lesional blood and performed TCR repertoire analysis for each skin lesion. In Case 10, we performed a skin biopsy from 3 isolated skin lesions (i.e., erythema, plaque, and tumor) (Fig. 4a–g). Few CD8^+^CD45RO^+^ cells, however, were detected in the plaque and tumor tissues in Case 10 (Fig. 4c,e). A characteristic repertoire pattern of CD8^+^CD45RO^+^ cells was detected in the different skin lesions of Case 10 (Fig. 4b,d,f). Case 15 showed a more skewed repertoire of the plaque tissue compared with an area of erythema from the same skin lesion (Fig. 4i,k). Although the lesional blood would not be a perfect representation of the cells in this lesion, we could indirectly show that different skin lesions have different clones of malignant cells. In other words, the TCR repertoire of CD8^+^CD45RO^+^ cells to malignant T cell antigens differed dependent on the skin lesion. Further studies with an increased number of cases are needed to investigate the details.

We performed the same procedure in psoriasis patients, and lesional blood in psoriasis exhibited a different pattern than lesional blood from MF (Fig. S1). The percentage of CD4^+^ T cells was higher in the lesional blood than in the peripheral blood (Fig. S1b). The CD4^+^ T cells of the lesional blood had a unique transcriptome, including *IL36B*, *FABP7*, *NLRC4,* and *FOS*. These genes lead inflammation and are detected in psoriatic lesions^42,43^. Interleukin (IL)-36 cytokines are a subgroup of the IL-1 cytokine family. IL-36B is highly detected in psoriatic lesions^44^. Chemokine receptors are G-protein coupled and T cells in lesional blood are induced by chemokines, therefore the top GO term is considered to “positively regulate the G protein coupled receptor signaling pathway”. The pattern of chemokine increases in psoriasis patients differed from that in MF patients (Fig. S1e). The pattern of chemokine increases in blood from psoriatic lesions are produced mainly by keratinocytes^45^. These chemokines are related to T cell and neutrophil trafficking^46,47^, and are specific to the pathogenesis of psoriasis. Therefore, we demonstrated that lesional blood obtained from psoriatic lesions can also reveal the psoriatic lesion environment.

In conclusion, we successfully developed a method for assessing the immune environment of CTCL from small amounts (drops) of lesional blood. Lesional blood drops contained sufficient numbers of isolated cells and amounts of sera for the assessment. The cells and sera isolated from lesional blood can also be used for further gene expression analysis of target cells. Our findings provide new insight into the pathogenesis of MF, a rare cutaneous lymphoma. The technique described in this study could be further applied to evaluate other skin inflammatory diseases, such as atopic dermatitis and psoriasis, as well as to assess the efficacy of treatments for these diseases.

## Methods

### Patients

We recruited 19 patients with MF (mean age: 67.10 ± 13.91 years; 9 women, 10 men) from the Department of Dermatology at the Nagoya City University. Exclusion criteria were: 1) age under 20 years, 2) HTLV-1 positive status, and 3) pregnant. The institutional review board of the Nagoya City University Graduate School of Medical Sciences approved the study (approval number: #60–18-0101). Written informed consent was obtained from the patients. All the experimental protocols adhered to relevant ethical guidelines for involving humans. The patients’ profiles are described in Table 1. Samples were obtained from the first 14 patients who visited our department from December 2018 to December 2019 and used for tissue staining, flow cytometry analyses, and chemokine assays. Some samples were subjected to RNA-seq (Cases 4 and 12) and TCR repertoire analyses (Cases 4, 10, 11, and 14). Samples obtained from Cases 15 through 19 were used for RNA-seq or TCR repertoire analyses (Table 1).

### Lesional blood collection

Skin biopsies were regularly obtained from the patients for diagnosis and assessment of treatment efficacy. After administering local anesthesia using lidocaine without epinephrine to the skin lesion, a punch biopsy was performed at a depth of 1 to 3 mm to avoid reaching the fatty layer. Oozing blood from the wounded area was collected as quickly as possible into an Eppendorf tube containing anticoagulant to avoid clotting. As anticoagulants, we used 5 μL of 100 U mL^−1^ heparin sodium (TERUMO) for flow cytometry analysis, and 3 μL of 0.5 mol L^−1^ EDTA (Invitrogen) for the RNA-seq and TCR repertoire analyses. Approximately 200 to 300 μL of blood was collected from each lesion area using a P20 Pipetman (Gilson). We also collected 30 to 50 μL of lesional blood without anticoagulant to obtain serum. After collecting the lesional blood, we performed another punch biopsy in the same lesion area, again at a sufficient depth to obtain a skin sample. Serum was collected and stored at − 80 °C until analysis. We collected peripheral blood from the patient’s arm and treated the peripheral blood in the same manner as the lesional blood.

### Flow cytometry

Peripheral and lesional blood samples were stained with antibodies specific for CD3 (clone SK7), CD4 (clone SK3), CD8 (clone SK1), CD45RA (clone L48), and CD45RO (clone UCHL-1) for 20 min at 23–25 °C. All antibodies used for flow cytometry were purchased from BD Biosciences. The antibodies for peripheral and lesional blood staining were hemolyzed with BD PharmLyse (BD Biosciences) for 15 min at room temperature, centrifuged, and then resuspended in staining buffer (BD Biosciences). Cells were analyzed on a FACS Verse flow cytometer (BD Biosciences), and data analysis was performed using the FlowJo software (FlowJo, version 10.6.2).

### Chemokine analysis

Serum levels of CCL2, CCL3, CCL4, CCL5, CCL11, CCL17, CCL20, CXCL5, CXCL8, CXCL9, CXCL10, and CXCL11 were determined using the Human Proinflammatory Chemokine Panel (BioLegend), and serum levels of CCL21 and CCL22 were determined using the AimPlex Premixed Multiplex kit (AimPlex Biosciences) according to the manufacturer’s instructions. All samples were read on a FACS Verse (BD Biosciences).

### Mass cytometric immunoassay

Blood cells were stained for mass cytometry after hemolysis using a Maxpar Human Peripheral Blood Phenotyping Panel kit (Fluidigm). Hemolyzed peripheral blood and lesional blood samples were resuspended in 1 mL phosphate-buffered saline and incubated for 5 min at room temperature with 1 mL of Cisplatin-108Pt (Fluidigm). The cells were washed using Maxpar Cell Staining Buffer (Fluidigm) and centrifuged, and the supernatant discarded; the pellets were then resuspended in 50 μL of the same buffer and 50 μL of a prepared cocktail of titrated Maxpar metal-conjugated antibodies was added (Fluidigm). After incubating for 15 min at room temperature, the cells were washed twice and fixed with 2% paraformaldehyde. The stained cells were analyzed by St. Luke’s MBL Corp using cytometry by time-of-flight. Mass cytometry data were analyzed using Cytobank (https://www.cytobank.org/).

### Cell sorting

CD4^+^CD45RO^+^ and CD8^+^CD45RO^+^ T cells were sorted using the BD FACSMelody Cell Sorter (BD Bioscience). The sorted T cells were collected for the TCR repertoire and RNA-seq analyses as described below.

### RNA-seq analysis

The CD4^+^CD45RO^+^ and CD8^+^CD45RO^+^ T cells were prepared as described above. The T cells were lysed with TRIzol reagent (Thermo Fisher Scientific) and stored at − 80 °C. The lysates were sent to Genewiz Japan Corp for RNA-seq and related analyses. In brief, RNA was extracted with chloroform/isopropanol and recovered from the supernatants using RNA Clean and Concentrator-5 columns (ZymoResearch) following the manufacturer’s instructions. The RNA purity was assessed with an Agilent 2100 Bioanalyzer. The RNA was subjected to library preparation with the TaKaRa SmartSeq Stranded Kit (Takara Bio) and sequenced with Illumina Hiseq (Illumina). Sequences were mapped to grch38 with HISAT2 (version 2.0.1). Differentially expressed genes were counted using the DESeq2 package in R (version 3.6.3). Up- and downregulated genes were defined as those (i) differentially expressed in peripheral and lesional blood cells with a *P*-value < 0.05, and (ii) having a greater than twofold change in the average normalized number of peripheral and lesional blood cells. Gene ontology analysis and enrichment analysis using the Jensen DISEASES dataset of differentially expressed genes was performed using the Enrichr webtool (https://maayanlab.cloud/Enrichr/) and Metascape (https://metascape.org/)^48–50^.

### TCR repertoire analysis

CD4^+^CD45RO^+^ and CD8^+^CD45RO^+^ T cells were prepared as described above. The T cells were lysed with Isogen-LS (NIPPON GENE) and stored at − 80 °C. The lysates were sent to Repertoire Genesis Inc. (Ibaraki, Japan) for next-generation sequencing, which was performed as previously described^51^. Briefly, total RNA was converted to complementary DNA (cDNA) with the SuperScript reverse transcriptase (Invitrogen). Double-stranded cDNA was synthesized and ligated with a 5′ adaptor oligonucleotide, then cut with the SphI restriction enzyme. Next, the double stranded cDNA was amplified through polymerase chain reaction using primers specific for the adaptor and TCRα constant region. The sequencing was performed with the Illumina MiSeq paired-end platform (2 × 300 bp). Data processing was performed with the repertoire analysis software developed by Repertoire Genesis Inc^52^. TCR sequences were assigned with a dataset of reference sequences from the international ImMunoGeneTics information system database (http://www.imgt.org). The percentage of sequence reads with TRAV, TRAJ, TRBV, and TRBJ genes was calculated. The Circos plots were produced using the Circos software package^53^. The Shannon–Weaver index shows the diversity and is defined as follows.

$$\mathrm{Shannon}-\mathrm{Weaver\, index }= -{\sum }_{\mathrm{i}-1}^{\mathrm{S}}\left(\frac{{\mathrm{n}}_{\mathrm{i}}}{\mathrm{N}}\mathrm{ln}\frac{{\mathrm{n}}_{\mathrm{i}}}{\mathrm{N}}\right)$$

### Immunohistochemistry and immunofluorescence staining

Biopsy skin specimens were fixed in 10% formalin and embedded in paraffin. Skin specimens were cut from the tissue block in 4-μm sections and stained with hematoxylin and eosin, anti-CD4 antibodies (Ab; clone EPR6855, Abcam), anti-CD8 Ab (clone C8/144B, Abcam), or anti-CD45RO Ab (clone UCH-L1, Absolute Antibody). Alexa488-conjugated goat anti-mouse IgG Ab (Invitrogen) and Alexa594-conjugated goat anti-rabbit IgG Ab (Invitrogen) were used as secondary antibodies. Fluorescent images were obtained using BZ-X810 (Keyence). The number of cells was determined with the cell count software BZ-H4C (Keyence).

### Statistics

Statistical analyses were performed using GraphPad Prism 7. All numerical data are summarized using mean ± standard deviation. Paired or unpaired Student t-tests were used to determine the significance of differences between groups, unless otherwise indicated in the figure legend. P-values < 0.05 were considered statistically significant.

## Supplementary Information

Supplementary Figure S1.

Supplementary Table S1.

Supplementary Table S2.

## Data Availability

All RNA-seq data sets have been deposited in the DNA Data Bank of Japan under accession numbers DRA010717 (http://www.ddbj.nig.ac.jp/intro-e.html). All data are available from the corresponding author upon reasonable request.
