# Supplementary material for: Determining the immune environment of cutaneous T-cell lymphoma lesions through the assessment of lesional blood drops
Source: Sci Rep. 2021 Oct 4;11:19629. doi: 10.1038/s41598-021-98804-0 (PMC8490448; doi:10.1038/s41598-021-98804-0)
Supplement: Supplementary file 1 — Supplementary Figure S1. [file 41598_2021_98804_MOESM1_ESM.pdf]

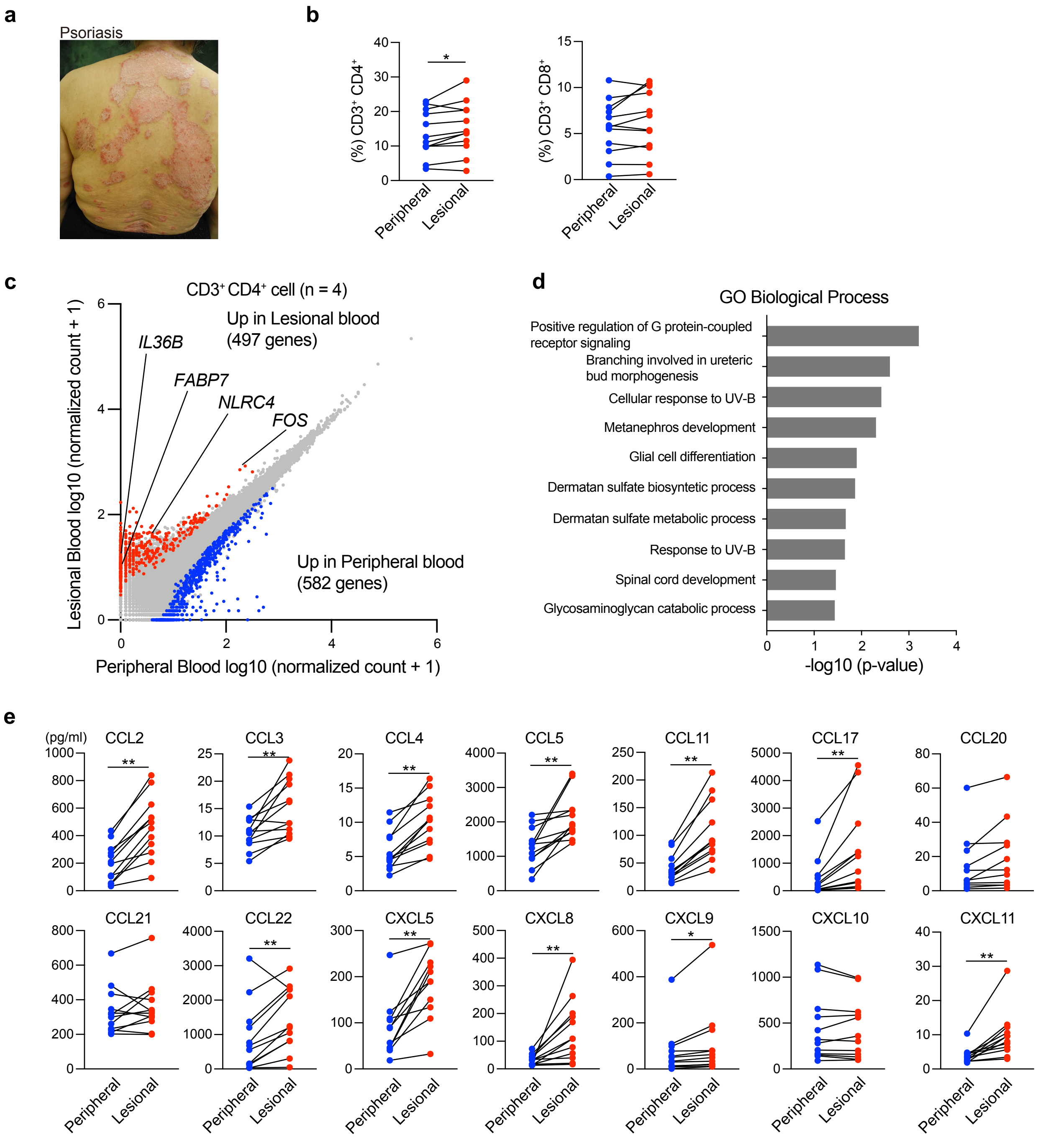

Figure S1. Assessment of lesional blood from psoriasis patients.
a) Clinical picture of psoriasis patient. b) Percentage of CD3+CD4+ and CD3+CD4+ cells in lesional blood and peripheral blood (n = 12). The percentage of CD3+CD4+ cells was higher in lesional blood than in peripheral blood. Paired t-test. ** P < 0.05. c) Gene expression analysis through RNA-seq in CD3+CD4+ cells of peripheral and lesional blood from 4 patients. Scatter plots show the expression values of every annotated gene. Blue and red dots indicate significant upregulation of CD3+CD4+ cells in peripheral and lesional blood, respectively. d) Ten top-ranked gene ontology terms of biologic processes for genes upregulated in CD3+CD4+ cells of lesional blood. e) Results of multiplex chemokine bead assay using sera from peripheral and lesional blood (n = 12). Paired t-test. * P < 0.05, ** P < 0.01.
