# Supplementary material for: Determining the immune environment of cutaneous T-cell lymphoma lesions through the assessment of lesional blood drops
Source: Sci Rep. 2021 Oct 4;11:19629. doi: 10.1038/s41598-021-98804-0 (PMC8490448; doi:10.1038/s41598-021-98804-0)
Supplement: Supplementary file 2 — Supplementary Table S1. [file 41598_2021_98804_MOESM2_ESM.docx]

Table S1. List of the differential expression genes shown in Fig 3.

| CD4^+^CD45RO^+^ cell | | | |
| --- | --- | --- | --- |
| Up in Lesional blood | | Up in Peripheral blood | |
| Gene ID | Symbol | Gene ID | Symbol |
| ENSG00000007174 | DNAH9 | ENSG00000006534 | ALDH3B1 |
| ENSG00000007171 | NOS2 | ENSG00000006747 | SCIN |
| ENSG00000007372 | PAX6 | ENSG00000007866 | TEAD3 |
| ENSG00000036448 | MYOM2 | ENSG00000008323 | PLEKHG6 |
| ENSG00000042781 | USH2A | ENSG00000016490 | CLCA1 |
| ENSG00000046604 | DSG2 | ENSG00000052344 | PRSS8 |
| ENSG00000070371 | CLTCL1 | ENSG00000060709 | RIMBP2 |
| ENSG00000079337 | RAPGEF3 | ENSG00000070985 | TRPM5 |
| ENSG00000090104 | RGS1 | ENSG00000072182 | ASIC4 |
| ENSG00000114315 | HES1 | ENSG00000073060 | SCARB1 |
| ENSG00000116147 | TNR | ENSG00000073756 | PTGS2 |
| ENSG00000118515 | SGK1 | ENSG00000074964 | ARHGEF10L |
| ENSG00000119715 | ESRRB | ENSG00000081923 | ATP8B1 |
| ENSG00000121039 | RDH10 | ENSG00000082482 | KCNK2 |
| ENSG00000121904 | CSMD2 | ENSG00000084710 | EFR3B |
| ENSG00000129116 | PALLD | ENSG00000088756 | ARHGAP28 |
| ENSG00000137571 | SLCO5A1 | ENSG00000092054 | MYH7 |
| ENSG00000141837 | CACNA1A | ENSG00000096696 | DSP |
| ENSG00000144460 | NYAP2 | ENSG00000099866 | MADCAM1 |
| ENSG00000145362 | ANK2 | ENSG00000100003 | SEC14L2 |
| ENSG00000146072 | TNFRSF21 | ENSG00000100884 | CPNE6 |
| ENSG00000146839 | ZAN | ENSG00000101203 | COL20A1 |
| ENSG00000147256 | ARHGAP36 | ENSG00000101213 | PTK6 |
| ENSG00000149256 | TENM4 | ENSG00000101333 | PLCB4 |
| ENSG00000149403 | GRIK4 | ENSG00000102802 | MEDAG |
| ENSG00000153234 | NR4A2 | ENSG00000103723 | AP3B2 |
| ENSG00000154237 | LRRK1 | ENSG00000105011 | ASF1B |
| ENSG00000161798 | AQP5 | ENSG00000105048 | TNNT1 |
| ENSG00000163251 | FZD5 | ENSG00000105278 | ZFR2 |
| ENSG00000163803 | PLB1 | ENSG00000105479 | CCDC114 |
| ENSG00000164393 | GPR111 | ENSG00000105492 | SIGLEC6 |
| ENSG00000170703 | TTLL6 | ENSG00000105509 | HAS1 |
| ENSG00000173567 | GPR113 | ENSG00000106410 | NOBOX |
| ENSG00000174705 | SH3PXD2B | ENSG00000106772 | PRUNE2 |
| ENSG00000175911 | AC127496.1 | ENSG00000108272 | DHRS11 |
| ENSG00000176083 | ZNF683 | ENSG00000108753 | HNF1B |
| ENSG00000176601 | MAP3K19 | ENSG00000109255 | NMU |
| ENSG00000178055 | PRSS42 | ENSG00000110786 | PTPN5 |
| ENSG00000183638 | RP1L1 | ENSG00000110975 | SYT10 |
| ENSG00000184144 | CNTN2 | ENSG00000111816 | FRK |
| ENSG00000187474 | FPR3 | ENSG00000113262 | GRM6 |
| ENSG00000196664 | TLR7 | ENSG00000113302 | IL12B |
| ENSG00000198216 | CACNA1E | ENSG00000113763 | UNC5A |
| ENSG00000198626 | RYR2 | ENSG00000115380 | EFEMP1 |
| ENSG00000215483 | LINC00598 | ENSG00000116176 | TPSG1 |
| ENSG00000223524 | REXO1L11P | ENSG00000117148 | ACTL8 |
| ENSG00000224796 | RPL32P1 | ENSG00000117266 | CDK18 |
| ENSG00000233785 | RP13-314C10.5 | ENSG00000118298 | CA14 |
| ENSG00000253767 | PCDHGA8 | ENSG00000118785 | SPP1 |
| ENSG00000259375 | RP11-815J21.2 | ENSG00000119782 | FKBP1B |
| ENSG00000260872 | RP11-680G24.5 | ENSG00000119862 | LGALSL |
|  |  | ENSG00000121207 | LRAT |
|  |  | ENSG00000121410 | A1BG |
|  |  | ENSG00000121933 | ADORA3 |
|  |  | ENSG00000122136 | OBP2A |
|  |  | ENSG00000124103 | FAM209A |
|  |  | ENSG00000128683 | GAD1 |
|  |  | ENSG00000129474 | AJUBA |
|  |  | ENSG00000130287 | NCAN |
|  |  | ENSG00000130377 | ACSBG2 |
|  |  | ENSG00000130477 | UNC13A |
|  |  | ENSG00000131095 | GFAP |
|  |  | ENSG00000131721 | RHOXF2 |
|  |  | ENSG00000132470 | ITGB4 |
|  |  | ENSG00000134072 | CAMK1 |
|  |  | ENSG00000134398 | ERN2 |
|  |  | ENSG00000135211 | TMEM60 |
|  |  | ENSG00000135253 | KCP |
|  |  | ENSG00000135409 | AMHR2 |
|  |  | ENSG00000135480 | KRT7 |
|  |  | ENSG00000137709 | POU2F3 |
|  |  | ENSG00000138083 | SIX3 |
|  |  | ENSG00000138606 | SHF |
|  |  | ENSG00000139354 | GAS2L3 |
|  |  | ENSG00000139572 | GPR84 |
|  |  | ENSG00000139874 | SSTR1 |
|  |  | ENSG00000140022 | STON2 |
|  |  | ENSG00000140986 | RPL3L |
|  |  | ENSG00000141338 | ABCA8 |
|  |  | ENSG00000141431 | ASXL3 |
|  |  | ENSG00000141497 | ZMYND15 |
|  |  | ENSG00000142156 | COL6A1 |
|  |  | ENSG00000142513 | ACPT |
|  |  | ENSG00000144452 | ABCA12 |
|  |  | ENSG00000144724 | PTPRG |
|  |  | ENSG00000145242 | EPHA5 |
|  |  | ENSG00000145626 | UGT3A1 |
|  |  | ENSG00000145920 | CPLX2 |
|  |  | ENSG00000146477 | SLC22A3 |
|  |  | ENSG00000146950 | SHROOM2 |
|  |  | ENSG00000147262 | GPR119 |
|  |  | ENSG00000147912 | FBXO10 |
|  |  | ENSG00000148156 | ACTL7B |
|  |  | ENSG00000148346 | LCN2 |
|  |  | ENSG00000149557 | FEZ1 |
|  |  | ENSG00000151276 | MAGI1 |
|  |  | ENSG00000151952 | TMEM132D |
|  |  | ENSG00000152932 | RAB3C |
|  |  | ENSG00000153956 | CACNA2D1 |
|  |  | ENSG00000154118 | JPH3 |
|  |  | ENSG00000155066 | PROM2 |
|  |  | ENSG00000155530 | LRGUK |
|  |  | ENSG00000155980 | KIF5A |
|  |  | ENSG00000156103 | MMP16 |
|  |  | ENSG00000157087 | ATP2B2 |
|  |  | ENSG00000157470 | FAM81A |
|  |  | ENSG00000157765 | SLC34A2 |
|  |  | ENSG00000157851 | DPYSL5 |
|  |  | ENSG00000158402 | CDC25C |
|  |  | ENSG00000161509 | GRIN2C |
|  |  | ENSG00000161664 | ASB16 |
|  |  | ENSG00000162595 | DIRAS3 |
|  |  | ENSG00000162643 | WDR63 |
|  |  | ENSG00000163739 | CXCL1 |
|  |  | ENSG00000164049 | FBXW12 |
|  |  | ENSG00000164197 | RNF180 |
|  |  | ENSG00000164509 | IL31RA |
|  |  | ENSG00000165092 | ALDH1A1 |
|  |  | ENSG00000165462 | PHOX2A |
|  |  | ENSG00000165480 | SKA3 |
|  |  | ENSG00000165862 | PNLIPRP2 |
|  |  | ENSG00000165972 | CCDC38 |
|  |  | ENSG00000166748 | AGBL1 |
|  |  | ENSG00000166816 | LDHD |
|  |  | ENSG00000166819 | PLIN1 |
|  |  | ENSG00000167554 | ZNF610 |
|  |  | ENSG00000167995 | BEST1 |
|  |  | ENSG00000168032 | ENTPD3 |
|  |  | ENSG00000168070 | C11orf85 |
|  |  | ENSG00000168079 | SCARA5 |
|  |  | ENSG00000168925 | CTRB1 |
|  |  | ENSG00000169255 | B3GALNT1 |
|  |  | ENSG00000169258 | GPRIN1 |
|  |  | ENSG00000169385 | RNASE2 |
|  |  | ENSG00000169429 | IL8 |
|  |  | ENSG00000169992 | NLGN2 |
|  |  | ENSG00000170231 | FABP6 |
|  |  | ENSG00000170537 | TMC7 |
|  |  | ENSG00000170624 | SGCD |
|  |  | ENSG00000170820 | FSHR |
|  |  | ENSG00000171004 | HS6ST2 |
|  |  | ENSG00000171121 | KCNMB3 |
|  |  | ENSG00000171747 | LGALS4 |
|  |  | ENSG00000171885 | AQP4 |
|  |  | ENSG00000172367 | PDZD3 |
|  |  | ENSG00000173110 | HSPA6 |
|  |  | ENSG00000173535 | TNFRSF10C |
|  |  | ENSG00000173898 | SPTBN2 |
|  |  | ENSG00000174460 | ZCCHC12 |
|  |  | ENSG00000174672 | BRSK2 |
|  |  | ENSG00000174945 | AMZ1 |
|  |  | ENSG00000175514 | GPR152 |
|  |  | ENSG00000176761 | ZNF285B |
|  |  | ENSG00000177112 | MRVI1-AS1 |
|  |  | ENSG00000177679 | SRRM3 |
|  |  | ENSG00000177694 | NAALADL2 |
|  |  | ENSG00000177992 | SPATA31E1 |
|  |  | ENSG00000178297 | TMPRSS9 |
|  |  | ENSG00000178922 | HYI |
|  |  | ENSG00000179111 | HES7 |
|  |  | ENSG00000179242 | CDH4 |
|  |  | ENSG00000181418 | DDN |
|  |  | ENSG00000181588 | MEX3D |
|  |  | ENSG00000181761 | OR8H3 |
|  |  | ENSG00000182575 | NXPH3 |
|  |  | ENSG00000182685 | BRICD5 |
|  |  | ENSG00000183018 | SPNS2 |
|  |  | ENSG00000183171 | RP11-49C9.2 |
|  |  | ENSG00000183251 | OR51B4 |
|  |  | ENSG00000183535 | COL18A1-AS1 |
|  |  | ENSG00000183770 | FOXL2 |
|  |  | ENSG00000183935 | HTR7P1 |
|  |  | ENSG00000184148 | SPRR4 |
|  |  | ENSG00000184599 | FAM19A3 |
|  |  | ENSG00000184956 | MUC6 |
|  |  | ENSG00000185271 | KLHL33 |
|  |  | ENSG00000185275 | CD24P4 |
|  |  | ENSG00000185633 | NDUFA4L2 |
|  |  | ENSG00000185842 | DNAH14 |
|  |  | ENSG00000186074 | CD300LF |
|  |  | ENSG00000186314 | PRELID2 |
|  |  | ENSG00000186715 | MST1L |
|  |  | ENSG00000187116 | LILRA5 |
|  |  | ENSG00000187791 | FAM205CP |
|  |  | ENSG00000188032 | C19orf67 |
|  |  | ENSG00000188368 | PRR19 |
|  |  | ENSG00000196811 | CHRNG |
|  |  | ENSG00000197013 | ZNF429 |
|  |  | ENSG00000197410 | DCHS2 |
|  |  | ENSG00000197702 | PARVA |
|  |  | ENSG00000198853 | RUSC2 |
|  |  | ENSG00000199927 | U3 |
|  |  | ENSG00000200295 | RNU6-527P |
|  |  | ENSG00000203783 | PRR9 |
|  |  | ENSG00000204053 | MYCLP1 |
|  |  | ENSG00000204539 | CDSN |
|  |  | ENSG00000205517 | RGL3 |
|  |  | ENSG00000205704 | LINC00634 |
|  |  | ENSG00000205795 | CYS1 |
|  |  | ENSG00000205832 | C16orf96 |
|  |  | ENSG00000211448 | DIO2 |
|  |  | ENSG00000211731 | TRBV5-7 |
|  |  | ENSG00000212930 | RP11-414H23.2 |
|  |  | ENSG00000213376 | GAPDHP71 |
|  |  | ENSG00000214107 | MAGEB1 |
|  |  | ENSG00000214353 | VAC14-AS1 |
|  |  | ENSG00000214694 | ARHGEF33 |
|  |  | ENSG00000214783 | POLR2J4 |
|  |  | ENSG00000214960 | ISPD |
|  |  | ENSG00000216522 | AC011718.1 |
|  |  | ENSG00000220575 | HTR5A-AS1 |
|  |  | ENSG00000221134 | AC007339.1 |
|  |  | ENSG00000222028 | PSMB11 |
|  |  | ENSG00000222036 | POTEG |
|  |  | ENSG00000223349 | KLF2P3 |
|  |  | ENSG00000223726 | CTA-929C8.7 |
|  |  | ENSG00000224155 | RP11-700P18.2 |
|  |  | ENSG00000224189 | HOXD-AS1 |
|  |  | ENSG00000224723 | GUSBP10 |
|  |  | ENSG00000225506 | CYP4A22-AS1 |
|  |  | ENSG00000225670 | CTA-134P22.2 |
|  |  | ENSG00000225706 | RP11-75C9.1 |
|  |  | ENSG00000225774 | SIRPAP1 |
|  |  | ENSG00000225826 | LINC00626 |
|  |  | ENSG00000226016 | RPL36AP55 |
|  |  | ENSG00000226051 | ZNF503-AS1 |
|  |  | ENSG00000226091 | LINC00937 |
|  |  | ENSG00000226281 | RP1-80N2.2 |
|  |  | ENSG00000226306 | NPY6R |
|  |  | ENSG00000227069 | CNN2P2 |
|  |  | ENSG00000227136 | LINC00595 |
|  |  | ENSG00000227303 | GS1-256O22.5 |
|  |  | ENSG00000227913 | KRT8P44 |
|  |  | ENSG00000228044 | RP4-781K5.4 |
|  |  | ENSG00000228075 | BOD1L2 |
|  |  | ENSG00000228133 | AC099684.1 |
|  |  | ENSG00000228158 | TLE1P1 |
|  |  | ENSG00000228335 | AC073063.10 |
|  |  | ENSG00000228521 | AC099552.3 |
|  |  | ENSG00000228554 | AC004837.5 |
|  |  | ENSG00000229243 | AC098973.1 |
|  |  | ENSG00000229326 | AC069154.4 |
|  |  | ENSG00000229849 | RP11-393K10.1 |
|  |  | ENSG00000230212 | AP000688.14 |
|  |  | ENSG00000230215 | EIF2B5-AS1 |
|  |  | ENSG00000230461 | PROX1-AS1 |
|  |  | ENSG00000230477 | AC005034.2 |
|  |  | ENSG00000230539 | AOAH-IT1 |
|  |  | ENSG00000230580 | AC021016.7 |
|  |  | ENSG00000230666 | CEACAM22P |
|  |  | ENSG00000230731 | RP11-478K15.6 |
|  |  | ENSG00000230753 | RP4-553F4.6 |
|  |  | ENSG00000230779 | RP1-140J1.4 |
|  |  | ENSG00000230990 | RP4-734C18.1 |
|  |  | ENSG00000230992 | FAM201B |
|  |  | ENSG00000231154 | MORF4L2-AS1 |
|  |  | ENSG00000231609 | AC009501.4 |
|  |  | ENSG00000231758 | AC092652.1 |
|  |  | ENSG00000231870 | KRT17P3 |
|  |  | ENSG00000232053 | AC009784.3 |
|  |  | ENSG00000232117 | LINC00384 |
|  |  | ENSG00000232204 | TET1P1 |
|  |  | ENSG00000232448 | RP11-416N4.1 |
|  |  | ENSG00000232553 | AC006026.10 |
|  |  | ENSG00000232903 | LINC01166 |
|  |  | ENSG00000233103 | RP11-524P6.1 |
|  |  | ENSG00000233271 | RP11-12C17.2 |
|  |  | ENSG00000233380 | RPS2P48 |
|  |  | ENSG00000233755 | RP4-799D16.1 |
|  |  | ENSG00000233825 | RP11-135A24.4 |
|  |  | ENSG00000234241 | RP4-775C13.1 |
|  |  | ENSG00000234282 | RP4-673D20.4 |
|  |  | ENSG00000234479 | AP1B1P1 |
|  |  | ENSG00000234647 | RP11-503C24.4 |
|  |  | ENSG00000234899 | SOX9-AS1 |
|  |  | ENSG00000235021 | RP11-439E19.7 |
|  |  | ENSG00000235102 | RP3-450M14.1 |
|  |  | ENSG00000235109 | ZSCAN31 |
|  |  | ENSG00000235387 | LINC00961 |
|  |  | ENSG00000235711 | ANKRD34C |
|  |  | ENSG00000236049 | AC104777.2 |
|  |  | ENSG00000236179 | RP13-221M14.4 |
|  |  | ENSG00000236618 | PITPNA-AS1 |
|  |  | ENSG00000236734 | GRIFIN |
|  |  | ENSG00000236780 | AC078941.1 |
|  |  | ENSG00000237990 | CNTN4-AS1 |
|  |  | ENSG00000238076 | MRPL48P1 |
|  |  | ENSG00000239210 | RPS26P55 |
|  |  | ENSG00000240184 | PCDHGC3 |
|  |  | ENSG00000240632 | SPATA31D5P |
|  |  | ENSG00000241529 | RN7SL767P |
|  |  | ENSG00000241595 | KRTAP9-4 |
|  |  | ENSG00000243064 | ABCC13 |
|  |  | ENSG00000243749 | ZMYM6NB |
|  |  | ENSG00000243957 | RN7SL647P |
|  |  | ENSG00000243959 | RN7SL684P |
|  |  | ENSG00000243974 | VTI1BP1 |
|  |  | ENSG00000244734 | HBB |
|  |  | ENSG00000245156 | RP11-867G23.3 |
|  |  | ENSG00000245870 | LINC00682 |
|  |  | ENSG00000248416 | RP11-1191J2.4 |
|  |  | ENSG00000248434 | RP11-553P9.2 |
|  |  | ENSG00000248492 | ZFAT-AS1 |
|  |  | ENSG00000248528 | CTC-458G6.2 |
|  |  | ENSG00000248647 | RP11-331K21.1 |
|  |  | ENSG00000248668 | OXCT1-AS1 |
|  |  | ENSG00000248881 | CTC-366B18.2 |
|  |  | ENSG00000248901 | AC008834.1 |
|  |  | ENSG00000249001 | RP11-742B18.1 |
|  |  | ENSG00000249464 | LINC01091 |
|  |  | ENSG00000249526 | CTB-35F21.1 |
|  |  | ENSG00000249534 | RP11-83C7.2 |
|  |  | ENSG00000249695 | RP11-598F7.4 |
|  |  | ENSG00000249960 | RP11-791G16.5 |
|  |  | ENSG00000250033 | SLC7A11-AS1 |
|  |  | ENSG00000250207 | AC079776.6 |
|  |  | ENSG00000250250 | CTD-2350J17.1 |
|  |  | ENSG00000250472 | TRIM36-IT1 |
|  |  | ENSG00000250846 | RP11-807H7.1 |
|  |  | ENSG00000250934 | RP11-71E19.1 |
|  |  | ENSG00000251095 | RP11-115D19.1 |
|  |  | ENSG00000251408 | RP11-586D19.2 |
|  |  | ENSG00000251676 | RP11-614F17.2 |
|  |  | ENSG00000252246 | RNA5SP92 |
|  |  | ENSG00000253147 | RP11-369E15.4 |
|  |  | ENSG00000253744 | AC025442.3 |
|  |  | ENSG00000253819 | LINC01151 |
|  |  | ENSG00000253899 | RP11-613H2.2 |
|  |  | ENSG00000253901 | RP11-122C21.1 |
|  |  | ENSG00000253958 | CLDN23 |
|  |  | ENSG00000254126 | CD8BP |
|  |  | ENSG00000254174 | IGHV1-12 |
|  |  | ENSG00000254221 | PCDHGB1 |
|  |  | ENSG00000255005 | RP11-90K17.2 |
|  |  | ENSG00000255085 | AF186192.5 |
|  |  | ENSG00000255161 | RP11-646J21.7 |
|  |  | ENSG00000255177 | RP11-532E4.2 |
|  |  | ENSG00000255213 | NPM1P35 |
|  |  | ENSG00000255363 | RP11-672A2.5 |
|  |  | ENSG00000255650 | FAM222A-AS1 |
|  |  | ENSG00000256995 | RP11-114G22.1 |
|  |  | ENSG00000257108 | NHLRC4 |
|  |  | ENSG00000258026 | RP11-543H12.1 |
|  |  | ENSG00000258083 | OR9A4 |
|  |  | ENSG00000258232 | RP11-161H23.5 |
|  |  | ENSG00000258384 | AC068831.6 |
|  |  | ENSG00000258729 | DKFZP434O1614 |
|  |  | ENSG00000258844 | RP11-259K15.2 |
|  |  | ENSG00000258916 | SPATA31E2P |
|  |  | ENSG00000259345 | RP11-624L4.1 |
|  |  | ENSG00000259370 | RP11-1069G10.1 |
|  |  | ENSG00000259728 | LINC00933 |
|  |  | ENSG00000260186 | RP11-481J2.2 |
|  |  | ENSG00000260196 | RP1-239B22.5 |
|  |  | ENSG00000260924 | AC004463.6 |
|  |  | ENSG00000261061 | RP11-303E16.2 |
|  |  | ENSG00000261127 | RP11-17M15.2 |
|  |  | ENSG00000261257 | RP11-673E11.2 |
|  |  | ENSG00000261285 | RP11-481E4.2 |
|  |  | ENSG00000261402 | RP11-378I6.1 |
|  |  | ENSG00000261664 | RP11-275F13.1 |
|  |  | ENSG00000261713 | SSTR5-AS1 |
|  |  | ENSG00000262061 | RP11-1260E13.4 |
|  |  | ENSG00000262899 | LA16c-360H6.3 |
|  |  | ENSG00000263218 | CTD-2561B21.7 |
|  |  | ENSG00000263413 | AL928742.1 |
|  |  | ENSG00000263932 | MIR4448 |
|  |  | ENSG00000263955 | RN7SL850P |
|  |  | ENSG00000264146 | AC021654.1 |
|  |  | ENSG00000265070 | AC006115.1 |
|  |  | ENSG00000266433 | TBC1D3P5 |
|  |  | ENSG00000266486 | FAM106CP |
|  |  | ENSG00000266751 | MIR3661 |
|  |  | ENSG00000267282 | CTB-129P6.4 |
|  |  | ENSG00000267304 | AC004637.1 |
|  |  | ENSG00000267414 | RP11-456K23.1 |
|  |  | ENSG00000267582 | CTD-3252C9.2 |
|  |  | ENSG00000267627 | RP11-905K4.1 |
|  |  | ENSG00000267750 | AC003102.3 |
|  |  | ENSG00000268455 | RP11-359H18.2 |
|  |  | ENSG00000268470 | DNAH17-AS1 |
|  |  | ENSG00000268964 | ERVV-2 |
|  |  | ENSG00000269473 | CTD-2619J13.19 |
|  |  | ENSG00000269481 | CTD-2521M24.6 |
|  |  | ENSG00000269635 | AC004257.1 |
|  |  | ENSG00000269926 | RP11-442H21.2 |
|  |  | ENSG00000269987 | RP3-430N8.11 |
|  |  | ENSG00000270314 | GS1-165B14.2 |
|  |  | ENSG00000270706 | CTD-2301A4.5 |
|  |  | ENSG00000270753 | RP11-693I21.1 |
|  |  | ENSG00000271207 | RP11-475J5.5 |
|  |  | ENSG00000271623 | RP11-435I10.5 |
|  |  | ENSG00000271811 | RP1-79C4.4 |
|  |  | ENSG00000272027 | RP11-529E15.1 |
|  |  | ENSG00000272808 | RP11-66B24.7 |
|  |  | ENSG00000272841 | RP3-428L16.2 |
|  |  | ENSG00000273001 | RP11-118K6.3 |
|  |  | ENSG00000273204 | RP4-549L20.3 |
|  |  | ENSG00000273230 | RP11-1246C19.1 |
|  |  | ENSG00000273321 | RP11-621L6.3 |
|  |  | ENSG00000273325 | RP1-90G24.11 |
|  |  | ENSG00000273492 | AP000230.1 |

| CD8^+^CD45RO^+^ cell | | | |
| --- | --- | --- | --- |
| Up in Lesional blood | | Up in Peripheral blood | |
| Gene ID | Symbol | Gene ID | Symbol |
| ENSG00000012504 | NR1H4 | ENSG00000007866 | TEAD3 |
| ENSG00000016082 | ISL1 | ENSG00000062370 | ZNF112 |
| ENSG00000016402 | IL20RA | ENSG00000066336 | SPI1 |
| ENSG00000019169 | MARCO | ENSG00000073756 | PTGS2 |
| ENSG00000049089 | COL9A2 | ENSG00000082929 | C4orf6 |
| ENSG00000056972 | TRAF3IP2 | ENSG00000083750 | RRAGB |
| ENSG00000068781 | STON1-GTF2A1L | ENSG00000090382 | LYZ |
| ENSG00000074211 | PPP2R2C | ENSG00000095303 | PTGS1 |
| ENSG00000079257 | LXN | ENSG00000099800 | TIMM13 |
| ENSG00000080200 | CRYBG3 | ENSG00000100319 | ZMAT5 |
| ENSG00000090861 | SEMA6A | ENSG00000100504 | PYGL |
| ENSG00000092421 | IGFALS | ENSG00000101336 | HCK |
| ENSG00000099769 | CTSG | ENSG00000101439 | CST3 |
| ENSG00000100448 | ASIP | ENSG00000102145 | GATA1 |
| ENSG00000101440 | PPP1R2P9 | ENSG00000103154 | NECAB2 |
| ENSG00000102055 | RP11-723C11 | ENSG00000104888 | SLC17A7 |
| ENSG00000103200 | A2M | ENSG00000104998 | IL27RA |
| ENSG00000104312 | RIPK2 | ENSG00000105514 | RAB3D |
| ENSG00000104892 | KLC3 | ENSG00000106384 | MOGAT3 |
| ENSG00000104951 | IL4I1 | ENSG00000109103 | UNC119 |
| ENSG00000105650 | PDE4C | ENSG00000109103 | PF4V1 |
| ENSG00000105767 | CADM4 | ENSG00000109272 | TBC1D9 |
| ENSG00000105948 | TTC26 | ENSG00000109436 | RNF141 |
| ENSG00000105982 | RNF32 | ENSG00000110315 | LIN7A |
| ENSG00000106823 | ECM2 | ENSG00000111052 | RERGL |
| ENSG00000107282 | APBA1 | ENSG00000112164 | GLP1R |
| ENSG00000109738 | GLRB | ENSG00000114631 | PODXL2 |
| ENSG00000109771 | LRP2BP | ENSG00000115183 | TANC1 |
| ENSG00000111701 | APOBEC1 | ENSG00000117791 | MARC2 |
| ENSG00000112333 | NR2E1 | ENSG00000120738 | EGR1 |
| ENSG00000112981 | NME5 | ENSG00000122642 | FKBP9 |
| ENSG00000115107 | STEAP3 | ENSG00000125538 | IL1B |
| ENSG00000118729 | CASQ2 | ENSG00000125804 | FAM182A |
| ENSG00000121075 | TBX4 | ENSG00000129152 | MYOD1 |
| ENSG00000121410 | GUCY1B2 | ENSG00000130545 | CRB3 |
| ENSG00000123201 | NLN | ENSG00000132205 | EMILIN2 |
| ENSG00000123213 | OBSL1 | ENSG00000132677 | RHBG |
| ENSG00000124006 | GDAP1L1 | ENSG00000133317 | LGALS12 |
| ENSG00000124194 | KCNK17 | ENSG00000136826 | KLF4 |
| ENSG00000124780 | TRIP10 | ENSG00000137440 | FGFBP1 |
| ENSG00000125733 | BCL2L12 | ENSG00000138685 | FGF2 |
| ENSG00000126453 | CDC42EP1 | ENSG00000143546 | S100A8 |
| ENSG00000128283 | MMP28 | ENSG00000144182 | LIPT1 |
| ENSG00000129270 | CDO1 | ENSG00000147588 | PMP2 |
| ENSG00000129596 | NCAN | ENSG00000149443 | C20orf78 |
| ENSG00000130287 | UNC13A | ENSG00000150681 | RGS18 |
| ENSG00000130477 | LRRC4B | ENSG00000152082 | MZT2B |
| ENSG00000131409 | RP11-798G7 | ENSG00000154146 | NRGN |
| ENSG00000132821 | POLR2A | ENSG00000155438 | NIFK |
| ENSG00000135116 | VSTM2L | ENSG00000156006 | NAT2 |
| ENSG00000135144 | HRK | ENSG00000156509 | FBXO43 |
| ENSG00000135406 | DTX1 | ENSG00000157600 | TMEM164 |
| ENSG00000136271 | PRPH | ENSG00000158955 | WNT9B |
| ENSG00000138798 | EGF | ENSG00000160683 | CXCR5 |
| ENSG00000143105 | KCNA10 | ENSG00000162040 | HS3ST6 |
| ENSG00000143631 | FLG | ENSG00000162383 | SLC1A7 |
| ENSG00000144339 | TMEFF2 | ENSG00000162493 | PDPN |
| ENSG00000144355 | DLX1 | ENSG00000163002 | NUP35 |
| ENSG00000144857 | BOC | ENSG00000163221 | S100A12 |
| ENSG00000145103 | ILDR1 | ENSG00000163281 | GNPDA2 |
| ENSG00000146378 | TAAR2 | ENSG00000163737 | PF4 |
| ENSG00000147883 | CDKN2B | ENSG00000164736 | SOX17 |
| ENSG00000149435 | GGTLC1 | ENSG00000165168 | CYBB |
| ENSG00000149654 | CDH22 | ENSG00000165204 | OR1K1 |
| ENSG00000151952 | TMEM132D | ENSG00000165264 | NDUFB6 |
| ENSG00000154153 | FAM134B | ENSG00000165724 | ZMYND19 |
| ENSG00000154768 | C17orf50 | ENSG00000165724 | STOX1 |
| ENSG00000154917 | RAB6B | ENSG00000165730 | RNASE7 |
| ENSG00000155330 | C16orf87 | ENSG00000165799 | ALKBH3 |
| ENSG00000156006 | FAM92A1P1 | ENSG00000166199 | LINC00483 |
| ENSG00000157021 | CDCP2 | ENSG00000167117 | SPINT2 |
| ENSG00000157211 | CREB3L1 | ENSG00000167642 | ZNF232 |
| ENSG00000157613 | C9orf43 | ENSG00000167840 | MIS12 |
| ENSG00000157653 | PAGE5 | ENSG00000167842 | OR5AU1 |
| ENSG00000158639 | LCN1 | ENSG00000169327 | PTAFR |
| ENSG00000160349 | LSP1P3 | ENSG00000169403 | RNASE6 |
| ENSG00000162685 | ATF3 | ENSG00000169413 | HOXD12 |
| ENSG00000162772 | NEUROD1 | ENSG00000170178 | TRH |
| ENSG00000162992 | KIF6 | ENSG00000171819 | ANGPTL7 |
| ENSG00000164627 | CCDC171 | ENSG00000172377 | OR9I1 |
| ENSG00000164989 | TTC8 | ENSG00000173137 | ADCK5 |
| ENSG00000165533 | C16orf71 | ENSG00000174016 | FAM46D |
| ENSG00000166246 | RBPMS2 | ENSG00000175707 | C1orf172 |
| ENSG00000166831 | IGF2 | ENSG00000176984 | AP000679 |
| ENSG00000167244 | ZNF229 | ENSG00000177971 | IMP3 |
| ENSG00000167383 | GPD1 | ENSG00000178695 | KCTD12 |
| ENSG00000167588 | ANGPTL4 | ENSG00000179168 | GGN |
| ENSG00000167772 | SHOX2 | ENSG00000179331 | RAB39A |
| ENSG00000168779 | TPTE2P5 | ENSG00000180712 | RP11-290F5 |
| ENSG00000168852 | APEX2 | ENSG00000180803 | RP11-467D10 |
| ENSG00000169188 | RSPH10B2 | ENSG00000181029 | TRAPPC5 |
| ENSG00000169402 | HTRA4 | ENSG00000181588 | MEX3D |
| ENSG00000169495 | KRT75 | ENSG00000182774 | RPS17L |
| ENSG00000170454 | DYDC1 | ENSG00000183032 | SLC25A21 |
| ENSG00000170788 | CEL | ENSG00000186272 | ZNF17 |
| ENSG00000170835 | PLAC1 | ENSG00000187175 | KRTAP12-1 |
| ENSG00000170965 | NRTN | ENSG00000187483 | SERPINA13P |
| ENSG00000171119 | OR2M4 | ENSG00000187566 | NHLRC1 |
| ENSG00000171180 | RNASE11 | ENSG00000188536 | HBA2 |
| ENSG00000171428 | TUBB8 | ENSG00000188660 | LINC00319 |
| ENSG00000173464 | PARL | ENSG00000188848 | BEND4 |
| ENSG00000173876 | OR51T1 | ENSG00000188906 | LRRK2 |
| ENSG00000175193 | MRVI1-AS1 | ENSG00000188992 | LIPI |
| ENSG00000175899 | RPL13AP3 | ENSG00000189143 | CLDN4 |
| ENSG00000176900 | GRM8 | ENSG00000196652 | ZKSCAN5 |
| ENSG00000177112 | SSC5D | ENSG00000197249 | SERPINA1 |
| ENSG00000177350 | C10orf91 | ENSG00000197681 | TBC1D3 |
| ENSG00000177350 | PRR26 | ENSG00000198298 | ZNF485 |
| ENSG00000179603 | PIPSL | ENSG00000198454 | C9orf141 |
| ENSG00000179954 | ANKRD62 | ENSG00000202078 | Y_RNA |
| ENSG00000180066 | PLAG1 | ENSG00000204420 | C6orf25 |
| ENSG00000180525 | ASB18 | ENSG00000204538 | PSORS1C2 |
| ENSG00000180764 | EPGN | ENSG00000204560 | DHX16 |
| ENSG00000181222 | AC004980 | ENSG00000204790 | CBWD6 |
| ENSG00000181626 | NATP | ENSG00000205025 | OR5G5P |
| ENSG00000181690 | ANKS1B | ENSG00000205097 | FRG2 |
| ENSG00000182177 | TEX38 | ENSG00000205359 | SLCO6A1 |
| ENSG00000182585 | FOXI2 | ENSG00000207837 | MIR517B |
| ENSG00000185040 | SAMD11 | ENSG00000208028 | MIR616 |
| ENSG00000185046 | ZFP69B | ENSG00000211891 | IGHE |
| ENSG00000186118 | FAM122A | ENSG00000212998 | RBM12B-AS1 |
| ENSG00000186766 | AC018892 | ENSG00000213333 | NPM1P50 |
| ENSG00000187079 | NAT1 | ENSG00000213478 | CFL1P2 |
| ENSG00000187634 | RP11-159F24 | ENSG00000214063 | TSPAN4 |
| ENSG00000187801 | NYX | ENSG00000214204 | HNRNPA1P43 |
| ENSG00000187866 | OTUD6A | ENSG00000214872 | SMTNL1 |
| ENSG00000188383 | PTPRT | ENSG00000217261 | POM121L4P |
| ENSG00000188850 | SPRR2B | ENSG00000217372 | TUBB4BP7 |
| ENSG00000188937 | LCA10 | ENSG00000218728 | KRT18P44 |
| ENSG00000189401 | OCLN | ENSG00000219135 | RP11-129H15 |
| ENSG00000196090 | C2CD4A | ENSG00000221990 | C5orf55 |
| ENSG00000196805 | ZNF536 | ENSG00000223343 | RP13-131K19 |
| ENSG00000196987 | TAT | ENSG00000223440 | RP11-555J4 |
| ENSG00000197822 | LRRC10 | ENSG00000223635 | RP4-613A2 |
| ENSG00000198535 | INPP5F | ENSG00000223638 | RFPL4A |
| ENSG00000198597 | MIR133B | ENSG00000224185 | SNX18P9 |
| ENSG00000198650 | Y_RNA | ENSG00000224447 | HIST1H1PS2 |
| ENSG00000198812 | RNA5SP204 | ENSG00000224545 | AC008264 |
| ENSG00000198825 | A1BG | ENSG00000224846 | RP1-90J20 |
| ENSG00000199080 | DDO | ENSG00000225471 | RP11-262D11 |
| ENSG00000201134 | AC005042 | ENSG00000225612 | AC099552 |
| ENSG00000201415 | CBY3 | ENSG00000225731 | AP001627 |
| ENSG00000203797 | CTD-3232M19 | ENSG00000225764 | LEPREL1-AS1 |
| ENSG00000204380 | FAM27E4 | ENSG00000225881 | AC009365 |
| ENSG00000204659 | ST8SIA6-AS1 | ENSG00000226330 | RP11-739N20 |
| ENSG00000204771 | TPTE2P6 | ENSG00000226469 | ADAM1B |
| ENSG00000204805 | AC005841 | ENSG00000226536 | SETP15 |
| ENSG00000204832 | CTD-2008L17 | ENSG00000226818 | SLC6A6P1 |
| ENSG00000205822 | RNU1-109P | ENSG00000226831 | MED15P3 |
| ENSG00000206044 | MIR127 | ENSG00000227115 | RP11-267C16 |
| ENSG00000206129 | MIR223 | ENSG00000227159 | DDX11L16 |
| ENSG00000206687 | RNU1-101P | ENSG00000228120 | AP001631 |
| ENSG00000207608 | KRTAP4-11 | ENSG00000228219 | NPM1P30 |
| ENSG00000207939 | TPM3P1 | ENSG00000229492 | AC004019 |
| ENSG00000212473 | AC104131 | ENSG00000229589 | ACVR2B-AS1 |
| ENSG00000212721 | AL591516 | ENSG00000229855 | CTC-546K23 |
| ENSG00000213050 | RPS7P8 | ENSG00000230111 | AC115283 |
| ENSG00000213115 | HMGB1P37 | ENSG00000230257 | NFE4 |
| ENSG00000213167 | POLD2P1 | ENSG00000230520 | RP11-533K11 |
| ENSG00000213183 | RPS10P2 | ENSG00000231280 | SALL4P6 |
| ENSG00000213467 | GNAT3 | ENSG00000231473 | LINC00441 |
| ENSG00000213730 | KRT18P13 | ENSG00000231575 | RP11-162K11 |
| ENSG00000213950 | NIFKP1 | ENSG00000231678 | RP11-339N8 |
| ENSG00000214415 | NUTM2D | ENSG00000231934 | RP4-610C12 |
| ENSG00000214417 | CAPN14 | ENSG00000232060 | RP11-255N24 |
| ENSG00000214434 | CDIPT-AS1 | ENSG00000232125 | DYTN |
| ENSG00000214562 | RP11-1036E20 | ENSG00000232401 | LINC00112 |
| ENSG00000214711 | MPRIPP1 | ENSG00000232815 | LINC00537 |
| ENSG00000214725 | DDX56 | ENSG00000232915 | AC097721 |
| ENSG00000214797 | GRID2IP | ENSG00000233167 | EEF1A1P26 |
| ENSG00000214820 | LINC01020 | ENSG00000233455 | RP4-667H12 |
| ENSG00000215045 | PPIAP1 | ENSG00000233614 | DDX11L10 |
| ENSG00000215231 | RP5-967N21 | ENSG00000233806 | AC131097 |
| ENSG00000215351 | GOLGA6L18 | ENSG00000233912 | AC026202 |
| ENSG00000215589 | RP1-290I10 | ENSG00000233997 | AP000475 |
| ENSG00000215749 | AC131097 | ENSG00000234323 | RP11-308N19 |
| ENSG00000216781 | FAM19A5 | ENSG00000234502 | FYTTD1P1 |
| ENSG00000216921 | RP1-172I22 | ENSG00000234816 | U91328 |
| ENSG00000219438 | RP1-59B16 | ENSG00000235323 | COTL1P2 |
| ENSG00000219784 | U3 | ENSG00000235475 | RP11-166O4 |
| ENSG00000220748 | MIR548T | ENSG00000235493 | AC092415 |
| ENSG00000221043 | AC013489 | ENSG00000235602 | POU5F1P3 |
| ENSG00000221296 | AL365361 | ENSG00000235625 | RP11-203P2 |
| ENSG00000221391 | AL354775 | ENSG00000235640 | AC092646 |
| ENSG00000221538 | AL031653 | ENSG00000236206 | RP11-306I1 |
| ENSG00000221685 | RN7SKP188 | ENSG00000236263 | RP11-263K19 |
| ENSG00000221726 | RN7SKP29 | ENSG00000236358 | RP5-827C21 |
| ENSG00000222721 | RN7SKP97 | ENSG00000236519 | AL773604 |
| ENSG00000222889 | RNU6-195P | ENSG00000236700 | LINC01010 |
| ENSG00000222898 | NEFHP1 | ENSG00000237000 | PTMAP6 |
| ENSG00000223284 | COX5BP8 | ENSG00000237206 | IMPDH1P4 |
| ENSG00000223489 | RP1-231P7P | ENSG00000237442 | HNRNPA1P57 |
| ENSG00000223794 | MCCD1P2 | ENSG00000238109 | AC004893 |
| ENSG00000224027 | LL22NC03-13G6 | ENSG00000238141 | BRWD1-AS1 |
| ENSG00000224312 | AC007182 | ENSG00000238247 | GS1-309P15 |
| ENSG00000224404 | RP11-54O15 | ENSG00000238390 | SNORA81 |
| ENSG00000224721 | A2MP1 | ENSG00000238892 | snoU13 |
| ENSG00000224764 | KRT18P39 | ENSG00000239210 | RPS26P55 |
| ENSG00000224791 | DUX4L9 | ENSG00000239345 | HNRNPA1P26 |
| ENSG00000224807 | U82695 | ENSG00000239998 | LILRA2 |
| ENSG00000224963 | AKR1B1P6 | ENSG00000240393 | RP11-768G7 |
| ENSG00000225212 | RFPL1S | ENSG00000240823 | RN7SL23P |
| ENSG00000225465 | AC018799 | ENSG00000241472 | PTPRG-AS1 |
| ENSG00000225539 | RP11-325P15 | ENSG00000241563 | CORT |
| ENSG00000225603 | RP11-127O4 | ENSG00000242391 | RP4-631H13 |
| ENSG00000225768 | AC009302 | ENSG00000242635 | RPS14P7 |
| ENSG00000226331 | RPS6P20 | ENSG00000243234 | CTD-2583A14 |
| ENSG00000226356 | ISCA2P1 | ENSG00000247134 | RP11-11N9 |
| ENSG00000226912 | ANHX | ENSG00000247473 | CARS-AS1 |
| ENSG00000227059 | OR2AS1P | ENSG00000247596 | TWF2 |
| ENSG00000227102 | ZNF717 | ENSG00000247728 | RP11-932O9 |
| ENSG00000227124 | AC073621 | ENSG00000248455 | RP11-321E2 |
| ENSG00000227158 | KIF9-AS1 | ENSG00000249252 | RP11-665G4 |
| ENSG00000227398 | SDAD1P3 | ENSG00000249697 | RP11-155L15 |
| ENSG00000227707 | VTI1BP4 | ENSG00000249887 | RP11-457P14 |
| ENSG00000227759 | AC069257 | ENSG00000250183 | RP11-96A1 |
| ENSG00000228028 | TEAD1 | ENSG00000250325 | IGBP1P4 |
| ENSG00000228221 | LINC00578 | ENSG00000250329 | KDELC1P1 |
| ENSG00000228326 | RP11-109G10 | ENSG00000250604 | RP11-597D13 |
| ENSG00000228513 | AC023271 | ENSG00000250896 | RNPS1P1 |
| ENSG00000228569 | AC073133 | ENSG00000251031 | CTC-367F4 |
| ENSG00000228626 | RP11-495P10 | ENSG00000251643 | RP11-91J3 |
| ENSG00000228714 | RP11-284G10 | ENSG00000252475 | RNU6-1332P |
| ENSG00000228888 | RP4-764O22 | ENSG00000252490 | RN7SKP66 |
| ENSG00000229154 | KCNQ5-AS1 | ENSG00000253706 | RP11-758M4 |
| ENSG00000229309 | RP11-452B18 | ENSG00000253824 | KB-173C10 |
| ENSG00000229657 | RP11-494K3 | ENSG00000253991 | KB-1562D12 |
| ENSG00000229951 | AC104695 | ENSG00000254144 | 7SK |
| ENSG00000229961 | RP11-71G12 | ENSG00000254194 | RP1-273G13 |
| ENSG00000230097 | RP11-460C6 | ENSG00000254468 | RP11-304M2 |
| ENSG00000230182 | RNF10P1 | ENSG00000254568 | RP11-664I21 |
| ENSG00000230246 | SPATA31C1 | ENSG00000254582 | PSMA2P1 |
| ENSG00000230318 | XRCC6P3 | ENSG00000254941 | RP11-677M14 |
| ENSG00000230372 | RP3-425P12 | ENSG00000255618 | RP11-357K6 |
| ENSG00000230415 | RP5-902P8 | ENSG00000255798 | RP11-817J15 |
| ENSG00000230474 | NAT2 | ENSG00000255980 | AP000439 |
| ENSG00000230815 | ATP6V1G1P7 | ENSG00000256101 | RP11-90D4 |
| ENSG00000231405 | RP11-49O14 | ENSG00000257389 | RP11-153F5 |
| ENSG00000231987 | RPL13AP | ENSG00000257766 | RP11-341G23 |
| ENSG00000232148 | CTA-992D9 | ENSG00000257872 | RP11-453D16 |
| ENSG00000232393 | RP5-898J17 | ENSG00000258302 | RP11-981P6 |
| ENSG00000232499 | FMO11P | ENSG00000258753 | RP11-794A8 |
| ENSG00000232540 | RPL5P6 | ENSG00000259049 | RP11-589M4 |
| ENSG00000232578 | RP11-512F24 | ENSG00000259125 | RP11-545N8 |
| ENSG00000232623 | RPL36P19 | ENSG00000259177 | RP11-154B12 |
| ENSG00000232678 | CTC-303L1 | ENSG00000259332 | ST20-MTHFS |
| ENSG00000232724 | AP000266 | ENSG00000259446 | RP11-489D6 |
| ENSG00000232846 | SPTLC1P4 | ENSG00000259539 | CTD-2651B20 |
| ENSG00000232875 | AC111186 | ENSG00000259684 | RP11-120K9 |
| ENSG00000233014 | SLC25A6P3 | ENSG00000260101 | RP11-568N6 |
| ENSG00000233026 | HMGN2P35 | ENSG00000260125 | AGBL1-AS1 |
| ENSG00000233148 | TBC1D3P7 | ENSG00000260205 | RP11-108B14 |
| ENSG00000233516 | AC026166 | ENSG00000260555 | RP11-728K20 |
| ENSG00000233544 | SYF2P2 | ENSG00000260579 | RP11-382A20 |
| ENSG00000233557 | RP11-388N2 | ENSG00000260810 | CTD-2547L24 |
| ENSG00000234034 | EIF3KP2 | ENSG00000260913 | RP11-243E13 |
| ENSG00000234139 | NFU1P2 | ENSG00000261242 | CTD-2302E22 |
| ENSG00000234207 | AP000959 | ENSG00000261280 | CTD-3105H18 |
| ENSG00000234457 | RP4-550H1 | ENSG00000261554 | RP11-810K23 |
| ENSG00000234509 | AC096570 | ENSG00000261644 | RP11-327F22 |
| ENSG00000234732 | AC006960 | ENSG00000261662 | RP5-1042I8 |
| ENSG00000234906 | AP000253 | ENSG00000261668 | RP11-50D9 |
| ENSG00000235023 | RPEP5 | ENSG00000261673 | RP11-328J14 |
| ENSG00000235098 | APOC2 | ENSG00000261752 | RP11-244B22 |
| ENSG00000235154 | AP001626 | ENSG00000261884 | CTC-479C5 |
| ENSG00000235268 | ANKRD65 | ENSG00000261916 | RP11-235E17 |
| ENSG00000235419 | CTA-280A3__B | ENSG00000262011 | AC003009 |
| ENSG00000235530 | KDM4E | ENSG00000262454 | RP11-65J21 |
| ENSG00000235559 | AC010149 | ENSG00000262681 | RP11-311F12 |
| ENSG00000235610 | AC087294 | ENSG00000262814 | MRPL12 |
| ENSG00000235616 | NOL5BP | ENSG00000262899 | LA16c-360H6 |
| ENSG00000235711 | AC013448 | ENSG00000263164 | RP11-333E1 |
| ENSG00000235888 | ST13P2 | ENSG00000263165 | RP11-810M2 |
| ENSG00000236056 | ANKRD34C | ENSG00000263657 | RP11-82O19 |
| ENSG00000236352 | AF064858 | ENSG00000264527 | WI2-1959D15 |
| ENSG00000236772 | GAPDHP14 | ENSG00000264792 | MIR4637 |
| ENSG00000236844 | AC005220 | ENSG00000264868 | CTB-167B5 |
| ENSG00000236932 | RP5-1184F4 | ENSG00000264939 | RP11-1109M24 |
| ENSG00000237111 | AC091633 | ENSG00000266371 | RP11-142O6 |
| ENSG00000237190 | CEACAMP8 | ENSG00000267244 | CTB-31O20 |
| ENSG00000237291 | IGHJ3P | ENSG00000267474 | CTC-548K16 |
| ENSG00000237436 | CDKN2AIPNL | ENSG00000267661 | RP11-820I16 |
| ENSG00000237483 | RP11-782C8 | ENSG00000268336 | SIGLEC20P |
| ENSG00000237640 | RP11-312B8 | ENSG00000268785 | RPL7P50 |
| ENSG00000237720 | RP11-423E7 | ENSG00000268947 | AD000684 |
| ENSG00000238033 | RP4-604G5 | ENSG00000269012 | KRT18P40 |
| ENSG00000238220 | AC011995 | ENSG00000270971 | REXO1L8P |
| ENSG00000238270 | AC002480 | ENSG00000271155 | RP11-435O5 |
| ENSG00000238965 | AP000275 | ENSG00000271620 | IGHV3OR16-7 |
| ENSG00000238976 | ACTBP4 | ENSG00000271681 | RP11-1259L22 |
| ENSG00000239397 | RP11-445J9 | ENSG00000271871 | AC005740 |
| ENSG00000239744 | RNA5SP351 | ENSG00000272009 | RP1-313I6 |
| ENSG00000239961 | AC004016 | ENSG00000272080 | MIR502 |
| ENSG00000239991 | RP11-101K23 | ENSG00000273258 | RP11-394I13 |
| ENSG00000240270 | RN7SL63P |  |  |
| ENSG00000240562 | LILRA4 |  |  |
| ENSG00000240922 | RP11-889D3 |  |  |
| ENSG00000241054 | RPL12P37 |  |  |
| ENSG00000241250 | RP11-59J16 |  |  |
| ENSG00000241335 | LSAMP-AS1 |  |  |
| ENSG00000241346 | RP5-1002M8 |  |  |
| ENSG00000241528 | RPL17P20 |  |  |
| ENSG00000241668 | RNA5-8S5 |  |  |
| ENSG00000242080 | RP11-379B18 |  |  |
| ENSG00000242419 | RP1-130H16 |  |  |
| ENSG00000243005 | AARS1 |  |  |
| ENSG00000243014 | RPL19P11 |  |  |
| ENSG00000243265 | CTC-550M4 |  |  |
| ENSG00000243440 | PCDHGC4 |  |  |
| ENSG00000243896 | RN7SL16P |  |  |
| ENSG00000243957 | PTMAP8 |  |  |
| ENSG00000244184 | RP11-1149O23 |  |  |
| ENSG00000244226 | AF165138 |  |  |
| ENSG00000246982 | OR2A7 |  |  |
| ENSG00000247081 | RN7SL647P |  |  |
| ENSG00000248673 | RP11-314A20 |  |  |
| ENSG00000248846 | ILF2P1 |  |  |
| ENSG00000248932 | RP1-179N16 |  |  |
| ENSG00000249053 | RP11-318M2 |  |  |
| ENSG00000249170 | CTC-419K13 |  |  |
| ENSG00000249228 | CTD-2016O11 |  |  |
| ENSG00000249337 | RP11-319G6 |  |  |
| ENSG00000249396 | RP11-419C19 |  |  |
| ENSG00000249564 | RP11-1J11 |  |  |
| ENSG00000249584 | RP11-769N22 |  |  |
| ENSG00000249770 | SNX18P25 |  |  |
| ENSG00000250076 | RP11-1C1 |  |  |
| ENSG00000250099 | RP11-390C19 |  |  |
| ENSG00000250221 | RP11-478P10 |  |  |
| ENSG00000250475 | MTND6P17 |  |  |
| ENSG00000250640 | RP11-141P6 |  |  |
| ENSG00000250947 | RP11-12P19 |  |  |
| ENSG00000250949 | KRT8P32 |  |  |
| ENSG00000251209 | RP11-312A15 |  |  |
| ENSG00000251297 | RP11-7O20 |  |  |
| ENSG00000251338 | TRPC7-AS2 |  |  |
| ENSG00000251373 | RP11-492L8 |  |  |
| ENSG00000251389 | LINC00923 |  |  |
| ENSG00000253199 | TUBB7P |  |  |
| ENSG00000253301 | AC006499 |  |  |
| ENSG00000253458 | RP11-68D16 |  |  |
| ENSG00000253516 | YTHDF1P1 |  |  |
| ENSG00000253542 | RP11-421P23 |  |  |
| ENSG00000253576 | RP11-513O17 |  |  |
| ENSG00000253670 | IGHVII-15-1 |  |  |
| ENSG00000253937 | HMGB1P41 |  |  |
| ENSG00000253943 | SDR16C6P |  |  |
| ENSG00000254092 | GS1-5L10 |  |  |
| ENSG00000254187 | RP11-429O2 |  |  |
| ENSG00000254258 | KRT18P37 |  |  |
| ENSG00000254285 | RP11-369E15 |  |  |
| ENSG00000254433 | CTB-78F1 |  |  |
| ENSG00000254677 | RP11-398H6 |  |  |
| ENSG00000254725 | KRT8P3 |  |  |
| ENSG00000254800 | RP11-677I18 |  |  |
| ENSG00000254951 | OSBPL9P2 |  |  |
| ENSG00000255005 | RP11-111A24 |  |  |
| ENSG00000255079 | RP11-163O19 |  |  |
| ENSG00000255145 | RP11-494M8 |  |  |
| ENSG00000255248 | RP11-90K17 |  |  |
| ENSG00000255396 | RP11-45A12 |  |  |
| ENSG00000255479 | RP11-60I3 |  |  |
| ENSG00000255558 | RP11-166D19 |  |  |
| ENSG00000255599 | AP000857 |  |  |
| ENSG00000255903 | RP11-672A2 |  |  |
| ENSG00000256069 | CTC-497E21 |  |  |
| ENSG00000256797 | AP000997 |  |  |
| ENSG00000257023 | RP11-136I14 |  |  |
| ENSG00000257108 | KLRF2 |  |  |
| ENSG00000257386 | RP11-268P4 |  |  |
| ENSG00000257398 | NHLRC4 |  |  |
| ENSG00000257446 | RP11-56G10 |  |  |
| ENSG00000257556 | RP11-554D14 |  |  |
| ENSG00000257568 | ZNF878 |  |  |
| ENSG00000257576 | RP11-44N21 |  |  |
| ENSG00000257761 | RP11-863H1 |  |  |
| ENSG00000257955 | RP11-153M3 |  |  |
| ENSG00000257956 | RP11-186F10 |  |  |
| ENSG00000257958 | RP1-228P16 |  |  |
| ENSG00000257989 | RP11-359M6 |  |  |
| ENSG00000258083 | RP11-25E2 |  |  |
| ENSG00000258120 | RP1-288H2 |  |  |
| ENSG00000258385 | OR9A4 |  |  |
| ENSG00000258466 | KRT128P |  |  |
| ENSG00000258472 | RP11-629F19 |  |  |
| ENSG00000258491 | RP11-1012A1 |  |  |
| ENSG00000258871 | RP11-192H23 |  |  |
| ENSG00000259228 | ZFP64P1 |  |  |
| ENSG00000259231 | RP3-514A23 |  |  |
| ENSG00000259240 | HNRNPA1P62 |  |  |
| ENSG00000259337 | CTD-2014N11 |  |  |
| ENSG00000259459 | RP11-108K3 |  |  |
| ENSG00000259488 | IGHV1OR15-2 |  |  |
| ENSG00000259543 | RP11-321G12 |  |  |
| ENSG00000259614 | RP11-154J22 |  |  |
| ENSG00000259775 | CTD-2034I4 |  |  |
| ENSG00000259800 | RP11-522B15 |  |  |
| ENSG00000259826 | RP11-45P15 |  |  |
| ENSG00000259871 | RP11-467D6 |  |  |
| ENSG00000259920 | CTA-363E6 |  |  |
| ENSG00000259946 | RP11-2E11 |  |  |
| ENSG00000260219 | RP11-490G2 |  |  |
| ENSG00000260318 | RP11-19N8 |  |  |
| ENSG00000260379 | RP11-347C12 |  |  |
| ENSG00000260441 | COX6CP1 |  |  |
| ENSG00000260477 | RP11-483K5 |  |  |
| ENSG00000260564 | RP11-96D1 |  |  |
| ENSG00000260602 | RP11-553E24 |  |  |
| ENSG00000260651 | RP11-403N16 |  |  |
| ENSG00000260672 | HMGN2P40 |  |  |
| ENSG00000260902 | AF213884 |  |  |
| ENSG00000261097 | RP11-1006G14 |  |  |
| ENSG00000261261 | RP11-95M5 |  |  |
| ENSG00000261318 | LINC00563 |  |  |
| ENSG00000261405 | RP11-297L17 |  |  |
| ENSG00000261420 | RP11-653J6 |  |  |
| ENSG00000261457 | RP11-23E10 |  |  |
| ENSG00000261566 | RP1-168L15 |  |  |
| ENSG00000262869 | AC002519 |  |  |
| ENSG00000263065 | CTD-2144E22 |  |  |
| ENSG00000263277 | CTD-2545H1 |  |  |
| ENSG00000264016 | AF001548 |  |  |
| ENSG00000264520 | RP11-293B20 |  |  |
| ENSG00000264573 | CTC-297N7 |  |  |
| ENSG00000264607 | RP4-777O23 |  |  |
| ENSG00000264954 | RN7SL15P |  |  |
| ENSG00000264954 | MIR3173 |  |  |
| ENSG00000265237 | RP11-214C8 |  |  |
| ENSG00000266242 | MIR3142 |  |  |
| ENSG00000266317 | NPM1P2 |  |  |
| ENSG00000266504 | GRAMD4P7 |  |  |
| ENSG00000266648 | RN7SL703P |  |  |
| ENSG00000267030 | RP11-112H10 |  |  |
| ENSG00000267091 | CTB-50L17 |  |  |
| ENSG00000267102 | RP11-820I16 |  |  |
| ENSG00000267440 | CTBP2P7 |  |  |
| ENSG00000267594 | RP11-686D22 |  |  |
| ENSG00000267610 | CTC-501O10 |  |  |
| ENSG00000267694 | CYP4F24P |  |  |
| ENSG00000267726 | AC007787 |  |  |
| ENSG00000267872 | RP11-691H4 |  |  |
| ENSG00000268208 | CTD-2526M8 |  |  |
| ENSG00000268402 | RP11-157B13 |  |  |
| ENSG00000268759 | AC008372 |  |  |
| ENSG00000268791 | AL031320 |  |  |
| ENSG00000268810 | AC135983 |  |  |
| ENSG00000268941 | AC093323 |  |  |
| ENSG00000270019 | AC007193 |  |  |
| ENSG00000270495 | MGC4294 |  |  |
| ENSG00000270957 | RP11-141B14 |  |  |
| ENSG00000270978 | CTD-2134P3 |  |  |
| ENSG00000271111 | RP11-1324A7 |  |  |
| ENSG00000271265 | RP11-54C4 |  |  |
| ENSG00000271419 | RP11-409K15 |  |  |
| ENSG00000271465 | RP11-230C9 |  |  |
| ENSG00000271500 | RP4-591B8 |  |  |
| ENSG00000271676 | SQSTM1P1 |  |  |
| ENSG00000271710 | RP11-288K12 |  |  |
| ENSG00000271784 | RP11-1E1 |  |  |
| ENSG00000271978 | RP11-312P12 |  |  |
| ENSG00000272256 | RP1-28H20 |  |  |
| ENSG00000272636 | RP11-428J1 |  |  |
| ENSG00000272825 | RP11-489E7 |  |  |
| ENSG00000272986 | DOC2B |  |  |
| ENSG00000273141 | LL21NC02-1C16 |  |  |
| ENSG00000273307 | RP11-46J23 |  |  |
| ENSG00000273361 | RP11-58E21 |  |  |
| ENSG00000286676 | RP11-378A13 |  |  |
