# Supplementary material for: Determining the immune environment of cutaneous T-cell lymphoma lesions through the assessment of lesional blood drops
Source: Sci Rep. 2021 Oct 4;11:19629. doi: 10.1038/s41598-021-98804-0 (PMC8490448; doi:10.1038/s41598-021-98804-0)
Supplement: Supplementary file 3 — Supplementary Table S2. [file 41598_2021_98804_MOESM3_ESM.docx]

Table S2. TP53 (rs1042522) and STAT3 polymorphism in CD4^+^CD45RO^+^ cells of 3 MF cases

| Patient | | TP53  c.C98G, p.P33R | STAT3  c.A1936T, p.N646Y | STAT3  c.A1480T, p.T494S |
| --- | --- | --- | --- | --- |
| Case12 | Peripheral | CC (100%) | AA (100%) | AA (100%) |
|  | Lesional |  | TT (100%) | AT (22%) |
| Case16 | Peripheral | CG (36%) | AA (100%) | AA (100%) |
|  | Lesional | CG (79%) |  |  |
| Case17 | Peripheral | GG (51%) | AA (100%) | AA (100%) |
|  | Lesional | GG (92%) |  |  |

These polymorphisms are detected by RNA-seq. Peripheral: Peripheral Blood, Lesional: Lesional Blood.
